# An Unsettled Promise: The Newborn Piglet Model of Neonatal Acute Respiratory Distress Syndrome (NARDS). Physiologic Data and Systematic Review

**DOI:** 10.3389/fphys.2019.01345

**Published:** 2019-10-30

**Authors:** Dietmar Spengler, Nele Rintz, Martin F. Krause

**Affiliations:** Department of Pediatrics, Universitätsklinikum Schleswig-Holstein, Kiel, Germany

**Keywords:** acute lung injury, pro-inflammatory pathways, immunosuppression, surfactant, mechanical ventilation, meconium aspiration model, lavage model, innate immunity

## Abstract

Despite great advances in mechanical ventilation and surfactant administration for the newborn infant with life-threatening respiratory failure no specific therapies are currently established to tackle major pro-inflammatory pathways. The susceptibility of the newborn infant with neonatal acute respiratory distress syndrome (NARDS) to exogenous surfactant is linked with a suppression of most of the immunologic responses by the innate immune system, however, additional corticosteroids applied in any severe pediatric lung disease with inflammatory background do not reduce morbidity or mortality and may even cause harm. Thus, the neonatal piglet model of acute lung injury serves as an excellent model to study respiratory failure and is the preferred animal model for reasons of availability, body size, similarities of porcine and human lung, robustness, and costs. In addition, similarities to the human toll-like receptor 4, the existence of intraalveolar macrophages, the sensitivity to lipopolysaccharide, and the production of nitric oxide make the piglet indispensable in anti-inflammatory research. Here we present the physiologic and immunologic data of newborn piglets from three trials involving acute lung injury secondary to repeated airway lavage (and others), mechanical ventilation, and a specific anti-inflammatory intervention via the intratracheal route using surfactant as a carrier substance. The physiologic data from many organ systems of the newborn piglet—but with preference on the lung—are presented here differentiating between baseline data from the uninjured piglet, the impact of acute lung injury on various parameters (24 h), and the follow up data after 72 h of mechanical ventilation. Data from the control group and the intervention groups are listed separately or combined. A systematic review of the newborn piglet meconium aspiration model and the repeated airway lavage model is finally presented. While many studies assessed lung injury scores, leukocyte infiltration, and protein/cytokine concentrations in bronchoalveolar fluid, a systematic approach to tackle major upstream pro-inflammatory pathways of the innate immune system is still in the fledgling stages. For the sake of newborn infants with life-threatening NARDS the newborn piglet model still is an unsettled promise offering many options to conquer neonatal physiology/immunology and to establish potent treatment modalities.

## Introduction

Respiratory failure is the leading cause of morbidity and mortality in newborn infants regardless of gestational age. Great advances in the construction of neonatal ventilators (continuous-flow) and in the development of assisted ventilation devices (e.g., invasive pressure-limited or volume-constant ventilation, continuous positive airway pressure breathing, nasal high-flow therapy) permitted to push back the thread of futile respiratory failure (Owen et al., 2017). Many years ago respiratory distress syndrome of the premature infant (IRDS) was attributed to a lack of surfactant production in the early stage of alveolar development of the immature lung (Farrell and Avery, 1975). However, respiratory failure of the term infant secondary to obvious damage of the lungs in the perinatal period, such as meconium, bile, and blood aspiration, lung hemorrhage, pneumonia, or severe chorioamnionitis and sepsis, leading to secondary impairment of surfactant function and surfactant amount, has not been officially defined before 2017 when the Montreux definition of neonatal ARDS (NARDS) was published (De Luca et al., 2017).

The Montreux definition of NARDS requires the following clinical conditions: respiratory failure of acute onset; exclusion of IRDS, transient tachypnea of the newborn (TTN), and congenital malformations of the lung; diffuse, bilateral, and irregular opacities or infiltrates by chest-Xray; lung edema of non-cardiac origin; and an oxygenation deficit expressed by the oxygenation index (OI = MAP ^*^ %O_2_/PaO_2_, with MAP = mean airway pressure) being mild (OI 4–8), moderate (OI 8–16), or severe (OI > 16).

Severe inflammation of the lung tissue in adult ARDS (ARDS) patients prompted researchers to investigate the effect of corticosteroids (Bernard et al., 1987; Steinberg et al., 2006; Needham et al., 2014) without being able to proof reduced mortality (except of the study by Meduri et al., 2007). Indeed, a pediatric study involving ARDS patients (PARDS) being subject to corticosteroid treatment showed increased mortality and less ventilator-free days (Yehya et al., 2015) whereas others (Drago et al., 2015; Kimura et al., 2016) could neither show clinical improvements by methylprednisolone infusions nor meaningful changes in plasma biomarker levels comparing methylprednisolone and placebo (e.g., MMP-8, Ang-2, sICAM-1, PAI-1, sRAGE).

In contrast to ARDS (Anzueto et al., 1996; Spragg et al., 2004; Kesecioglu et al., 2009; Willson et al., 2015), NARDS (Lotze et al., 1998) and PARDS (Herting et al., 2002; Möller et al., 2003; Willson et al., 2005) patients profit from their susceptibility to surfactant treatment. As surfactant is able to mitigate many components of lung inflammation (Kunzmann et al., 2013) its use may be universally indicated together with adjuncts specifically tackling pro-inflammatory pathways being central for lung inflammation. Thus, the pharmacologic armamentarium in the treatment of NARDS appears to be more variable and may be applied more individually than the classical immune-suppressive means in respiratory disease of children (i.e., corticosteroids) (de Benedictis and Bush, 2012).

The identification of major pro-inflammatory pathways [by the analysis of serum or broncho-alveolar lavage fluid (BALF)] causing respiratory failure in NARDS/PARDS has so far brought preliminary results only: De Luca et al. identified secretory phospholipase A2 secreted by alveolar macrophages as the main reason for surfactant degradation (De Luca et al., 2008) whereas in PARDS the analysis of serum Ang-2 and vWF yielded equivocal results (Kimura et al., 2016; Zinter et al., 2016), and the analysis of interleukins, IFN, MCP-1, G-CSF, and MMP-8 did not reveal any pathway-typical patterns (Kimura et al., 2016; Schwingshackl et al., 2016). As an example of ambiguity the study by Dahmer et al. (2018) assessing the role of the naturally occurring IL-1 (interleukin-1) receptor antagonist in the augmentation of PARDS is listed here which underlines the high complexity of natural inflammation and anti-inflammation for the disease process. As to surfactant composition, a decrease in saturated phosphatidylcholine (PC) and an increase in unsaturated PC combined with almost stable concentrations of the four surfactant proteins (SP), however an increase in SP-B as a parameter of capillary leakage, was found in children with a maximum OI of 12 (Todd et al., 2010).

In an attempt to better characterize and tackle major pro-inflammatory pathways in NARDS the neonatal piglet is the animal model of choice for reasons of availability, size, similarities of porcine and human lung, robustness, and costs. In addition, the pig's hypervariable region (HVR) of the toll-like receptor 4 shows high identity (and many nucleotide polymorphisms) with the human TLR4 HVR (Palermo et al., 2009), they are equipped with pulmonary intravascular macrophages, and show LPS sensitivity and NO production comparable to humans (Matute-Bello et al., 2008). To prove the advantages of this translational neonatal piglet model of NARDS, the physiologic data (with emphasis on lung function) from three experiments of our group are summarized here. In addition, the systematic review addresses different models of acute lung injury with respiratory failure in neonatal piglets, describes major pro-inflammatory pathways by the analysis of serum, BALF, and lung tissue, and highlights effective experimental interventions by anti-inflammatory substances.

## Methods

### Piglet Studies and Systematic Review: Data Sources and Searching

A compilation of data from three NARDS studies (von Bismarck et al., 2008; Preuß et al., 2012b; Spengler et al., 2018) was used to describe basic physiologic parameters and major inflammatory pathways of the neonatal porcine lung. The studies were approved by the local Ethics Committee for Animal Research at the Ministry of Energy, Agriculture, the Environment, Nature and Digitalization of the federal state of Schleswig-Holstein in accord with the current European directive on the protection of animals used for scientific purposes. Corresponding physiologic parameters from human neonates are provided as a comparison if available and deemed necessary.

In addition a systematic review on major inflammatory pathways following single-hit or multiple-hit acute lung injury in newborn piglets was conducted using PubMed and Google Scholar databases in search of the terms “newborn piglet” combined with “(acute) lung injury,” “mechanical ventilation,” “respiratory failure,” “lung inflammation,” “meconium aspiration,” “airway lavage,” and “lipopolysaccharide/endotoxin.” Reference lists and relevant reviews were also checked manually to recruit potentially eligible studies. Pulmonary physiology data and all data assessing inflammatory reactions secondary to acute lung injury protocols or specific interventions were extracted and reported.

### Neonatal Piglets, Mechanical Ventilation, Lung Injury Protocols, Interventions, and Statistics

The study population was newborn piglets between day 2 and 6 of life and of either sex that were taken from their mother sows without any period of fasting. Genetic variability was assured by the use of mixed country breed (descendants of Danish Landrace) piglets. Their average weight of 2.5 kg allowed to apply the standard equipment of an average neonatal intensive care unit for instrumentation, maintenance and interventions. The number of piglets included into the data analyses were 22 in study 1 (von Bismarck et al., 2008), 29 in study 2 (Preuß et al., 2012b), and 59 in study 3 (Spengler et al., 2018).

Adequate analgesia/sedation was provided by continuous infusions of ketamine (5 mg/kg/h), midazolam (0.5 mg/kg/h), and vecuronium bromide (0.8 mg/kg/h) throughout the whole study period of 24 h (study 1) or 72 h (studies 2 and 3). Nutritional support was provided via a nasogastric tube with 6^*^25 ml/kg/d specialized milk designed for piglets (Babygold, Hamburger Leistungsfutter). Body temperature of 38–39°C was maintained by positioning the piglets on a homeothermic blanket (Harvard Apparatus) and applying a rectal probe with the servo-control mode.

All piglets received mechanical ventilation via an orally inserted 3.5 mm endotracheal double lumen tube. Continuous-flow pressure-limited neonatal ventilators (Babylog 1, Dräger) were used with the following initial settings: PEEP = 6 mbar, inspiratory time = 0.5 s, *f* = 25/min, FiO_2_ = 0.5, PIP adjusted to maintain a tidal volume = 7 ml/kg as measured by NVM-1 (Bear) throughout the study. To avoid hypo-/hyperventilation and hypoxemia/hyperoxemia, f and FiO_2_ were regularly adjusted according to the results of arterial blood gas analyses. An oxygenation index (OI: MAP ^*^ %O_2_/PaO_2_, with MAP = mean airway pressure) and a ventilation efficiency index (VEI: 3800/PIP-PEEP ^*^f ^*^ PaCO_2_) were calculated from the parameters of the ventilator and the results of the arterial blood gas analysis. Functional residual capacity (FRC, ml/kg), the alveolar portion of the tidal volume (V_A_, ml), tidal volume (V_T_, ml) (specific) compliance of the respiratory system (sC_rs_, ml/mbar/kg), and resistance of the respiratory system (R_rs_, mbar/l^*^s) were assessed by the nitrogen washout method for lung volumes, and the single breath least-squares method for lung mechanics.

Hemodynamic monitoring was provided by PiCCO plus monitors (Pulsion) yielding a continuous monitoring of heart rate (HR), blood pressure (BP), heart index (HI), peripheral vascular resistance (SVRI), stroke volume variation (SVV), and extra-vascular lung water index (EVLWI).

Urine output was monitored continuously by the insertion of a suprapubic bladder catheter.

Two different lung injury protocols were used: in study 1, lung injury was provided by repeated airway lavage with warmed normal saline (30 ml/kg) until the PaO_2_ was ~100 mmHg and stayed at that level for at least 20 min (single-hit lung injury). In studies 2 and 3, three consecutive lung injury protocols were carried out of which the first one was repeated airway lavage as described above, followed by a 2 h period of injurious ventilation (by the use of a V_T_ = 15 ml/kg and PEEP = 0 mbar) 24 h later, and by the endotracheal instillation of 2.5 mg LPS (*E. coli* serotype O127:B8; Sigma-Aldrich) 48 h later (triple-hit lung injury).

Next to the control groups (C) subject to an air bolus only, the piglets received surfactant (poractant alfa, Curosurf, Chiesi) at a dosage of 1^*^100 mg/kg (study 1) or 3^*^50 (200) mg/kg every 24 h apart (studies 2 and 3) as an intervention. In several intervention groups the surfactant was “fortified” by additional immune-suppressive agents: imipramine 5 mg admixed to surfactant (study 1), D-*myo*-inositol-1,2,6-trisphosphate 2/2.5 mg (Cayman) (studies 2 and 3), *myo*-inositol 40 mg (Sigma-Aldrich) (study 2), phosphatidylinositol-3,5-bisphosphate 2.5 mg(Cayman) (study 3), palmitoyl-oleoyl-phosphatidylglycerol 7.5 mg (Avanti) (study 3), and dioleoyl-phosphatidylglycerol 7.5 mg (Avanti) (study 3). In this analysis the data of all intervention groups from one study are combined as the treatment group (T); the combination of C and T is reported as the total group in the tables to point out deviations from means and to prove the stability of the model. Study 3 also analyzed a group of piglets not being subject to sedation and mechanical ventilation that is reported as healthy controls (HC).

Next to the assessment of physiologic parameters, a variety of specific pulmonary parameters of the immune response to single-hit/triple-hit lung injury were performed by the use of lung sections (e.g., histology), lung homogenates (e.g., acid sphingomyelinase activity), and broncho-alveolar lavage fluid (BALF, e.g., cell differentials). For further details we refer to the detailed description of all applied methods in the methods sections of the referenced publications (von Bismarck et al., 2008; Preuß et al., 2012b; Spengler et al., 2018).

For repeated-measures data the two-way mixed ANOVA was used to determine whether there were differences of an independent variable [between subject factor: control (C), treatment (T), overall (O)] over time (within subject factor: baseline, 24, 48, 72 h). A normal distribution of the independent variable was assessed by Shapiro-Wilk's test (*p* > 0.05). Equality of error variances using Levene's test and equality of covariance matrices by Box's *M* test was carried out for every parameter; in case of heteroscedasticity data were transformed by the Box-Cox transformation before analysis. Mauchly's test of sphericity was performed on every parameter to check for significant two-way interaction (*p* < 0.05). The within subject factor and the interaction (within subject factor ^*^ between subject factor) were calculated by Greenhouse-Geisser correction in case the estimated epsilon was <0.75. The main effect of the between subject factor (group) on the independent variable was considered statistically significant in case of *p* < 0.05. Single data sets were checked for deviations from normality using the Shapiro-Wilk's test (*p* > 0.05). Normally distributed data were analyzed by unpaired *t*-tests, and non-parametric data by Mann-Whitney *U* tests. All data are presented as means ± SD. The analyses were performed by SPSS version 24 (IBM, Ehningen, Germany).

### Systematic Review: Study Selection, Data Extraction, and Assessment of Risk of Bias

Two authors (DS and NR) independently screened the titles provided by the combination of different search terms indicated above. The inclusion criteria were: studies published in English within the last 30 years following peer-review, studies reporting information on NARDS in neonatal piglets following distinct experimental lung injury protocols, studies reporting on major inflammatory pathways and their mediators. Publications were excluded if they did not report on a setting of invasive mechanical ventilation with at least one acute lung injury protocol, and if no adequate control group was presented. The quality of studies was independently evaluated by the two authors using the Quality Assessment Tool for Case-Control Studies by the National Heart, Lung, and Blood Institute (NHLBI)^1^.

## Results and Discussion

### Circulation

The cardiovascular stability was challenged in the context of direct and indirect manipulations of heart, systemic, and pulmonary circulation. In addition, the possible pharmacologic effects of sedatives/analgetics must be taken into account. For a sufficient stability of the circulation some drug classes, such as barbiturates and opiods seem to be less suited because of their negative inotropic action on the myocardium. In models covering more than 12 h of mechanical ventilation a cumulative effect and a progressive decline in HI and SVRI can be observed. As sufficient analgesia is paramount in any model opioids should be used for instrumentation and for all kinds of painful procedures, however, for long time sedation and analgesia ketamine (in combination with low dose benzodiazepine) seems to be more apt because of its positive inotropic effect even in the presence of muscular blockade.

The combined effects of the triple-hit lung injury protocol (repeated airway lavage, injurious ventilation, and endotracheal endotoxin installation) on cardiovascular parameters are shown in Table 1 covering a time window of 72 h (Spengler et al., 2018). The cardiovascular function is characterized by high stability in heart rate (HR), systolic and diastolic blood pressure (S/DBP), heart index (HI), systemic vascular resistances index (SVRI), intrathoracic blood volume index (ITBI), stroke volume index (SVI), and stroke volume variation (SVV) over 72 h of invasive monitoring despite statistically significant changes in DBP, SVRI, SVI, and SVV (time) and HR (time^*^group) (Table 1). However, no single parameter shows a continuously increasing or decreasing trend. We observed progressing blood pressure instability combined with increasing SVRI and decreasing HI in only 5/67 (7.5%) piglets, a reason for drop-out in this model.

**Table 1:** Circulation.

		**Baseline**	**24 h**	**48 h**	**72 h**	**Sphericity**	**Time**	Time*group	**Group**
HR (bpm)	Total	181 ± 25	175 ± 27	158 ± 24	156 ± 26	0.04	0.35	0.01	0.72
	Control	177 ± 29	182 ± 31	157 ± 20	146 ± 31				
	Treat	182 ± 25	174 ± 26	158 ± 25	158 ± 25				
SBP (mmHg)	Total	99 ± 15	89 ± 12	99 ± 15	97 ± 12	0.47	0.79	0.45	0.39
	Control	100 ± 15	86 ± 13	98 ± 16	92 ± 11				
	Treat	99 ± 15	90 ± 11	99 ± 15	98 ± 12				
DBP (mmHg)	Total	56 ± 8	44 ± 7	52 ± 8	49 ± 9	0.02	0.00	0.17	0.74
	Control	59 ± 6	45 ± 7	51 ± 7	45 ± 6				
	Treat	55 ± 9	44 ± 7	52 ± 8	50 ± 10				
HI (l/min/m^2^)	Total	3.9 ± 0.7	3.8 ± 0.9	3.8 ± 0.8	4.0 ± 0.9	0.52	0.21	0.37	0.58
	Control	4.1 ± 0.9	3.6 ± 1.0	3.9 ± 0.9	4.3 ± 1.0				
	Treat	3.8 ± 0.7	3.9 ± 0.8	3.8 ± 0.8	3.9 ± 0.9				
SVRI (dyne*sec*cm^−5^*m^2^)	Total	1,452 ± 281	1,285 ± 299	1,476 ± 271	1,362 ± 369	0.89	0.00	0.90	0.48
	Control	1,425 ± 208	1,187 ± 370	1,418 ± 361	1,307 ± 367				
	Treat	1,472 ± 259	1,309 ± 295	1,469 ± 257	1,333 ± 340				
ITBI (ml/m^2^)	Total	276 ± 56	308 ± 117	295 ± 68	298 ± 81	0.00	0.49	0.88	0.46
	Control	300 ± 18	323 ± 75	318 ± 58	331 ± 77				
	Treat	269 ± 62	305 ± 128	288 ± 71	289 ± 83				
SVI (ml/m^2^)	Total	23.5 ± 6.6	22.7 ± 6.2	25.0 ± 6.1	26.1 ± 7.1	0.16	0.00	0.09	0.50
	Control	27.6 ± 11.1	21.5 ± 6.1	23.7 ± 6.8	28.0 ± 5.9				
	Treat	22.7 ± 5.6	22.7 ± 5.0	24.4 ± 5.2	25.9 ± 7.1				
SVV (%)	Total	12.4 ± 3.8	9.6 ± 3.1	10.1 ± 3.9	8.8 ± 3.5	0.34	0.00	0.58	0.67
	Control	12.5 ± 4.2	10.6 ± 1.8	9.6 ± 4.4	7.1 ± 3.2				
	Treat	12.4 ± 3.9	9.4 ± 3.3	10.2 ± 3.8	9.1 ± 3.5				

*HR, heart rate; SBP, systolic blood pressure; DBP, diastolic blood pressure; HI, heart index; SVRI, systemic vascular resistance index; ITBI, intrathoracic blood volume index; SVI, stroke volume index; SVV, stroke volume variation*.

*Data are mean ± SD. Number of piglets included: total = 51, control = 8, treat(ment) = 43. Comparisons by two-way mixed ANOVA for repeated measures data*.

Data of circulatory parameters in the non-anesthetized piglet have been published by Eisenhauer et al. (1994) studying chronically instrumented neonatal piglets being individually raised and fed. The heart rate of 187 ± 28 bpm and the mean blood pressure of 66 ± 4 mm Hg are very close to the values obtained in our piglets at baseline being subject to anesthesia and mechanical ventilation, suggesting only minor influences of ketamine/midazolam/vecuronium bromide given as continuous drips on hemodynamic function. This is supported by the data from 5 to 7 days old piglets being subject to anesthesia with halothane and invasive blood pressure monitoring yielding values for SBP of 89 mmHg (CI 84–99) and DBP of 54 mmHg (51–60) (Voss et al., 2004). Using the thermodilution technique HI was 4.04–4.38 ± 1.23–1.42 l/min/m^2^, and the SVI 20.4/20.4 ± 5.7–9.5 ml/m^2^ in 13 days old piglets (Gibson et al., 1994), and ITBI 230 ± 76 ml/m^2^ in 1–3 days old piglets (Silvera et al., 2011).

The data of 90 healthy human neonates on day 3 of life assessed by ultrasonic cardiac output monitoring yielded the following results: HR 119 ± 12 bpm, SBP 73 ± 4 mmHg, DBP 39 ± 5 mmHg, HI 3.0 ± 0.6 l/min/m^2^, SVRI 1,403 ± 291 dyne^*^sec^*^cm^−5*^m^2^, and SVI 25.3 ± 5.1 ml/m^2^ (He et al., 2011). By the thermodilution technique in human newborns after arterial switch procedure due to transposition of the great arteries, CI was 4.0 ± 0.6 l/min/m^2^, SVRI 1,150 ± 295 dyne^*^sec^*^cm^−5*^m^2^, ITBI 489 ± 125 ml/m^2^, and SVI 33.1 ± 4.3 ml/m^2^ (Székely et al., 2011). While these latter data are probably not representative for healthy human newborns, there are obvious differences in circulation between porcine and human newborns: the porcine circulation generates a significant higher S/DBP level due to a higher HR and HI whereas SVRI is comparable to human values. In view of similar heart/body weight relationships [porcine: 0.89 ± 0.06% (Miles et al., 2012), 0.69 ± 0.02% (Amdi et al., 2013), 0.70 ± 0.06 (Farmer et al., 2016); human: 0.62–0.75 ± 0.35–0.50 (Corrèa et al., 2014)], an important prerequisite for circulatory stability, the piglet model excels over rodent animal models.

### Electrolytes and Renal Function

We observed significant (however clinically irrelevant) time-dependent changes in electrolytes, creatinine, and GOT (Table 2). Plasma Na (143 ± 5 mmol/l, 138 ± 3) and K (4.4 ± 0.8 mmol/l, 4.2 ± 0.4) concentrations in 2–5 days old piglets were comparable to our results (Parker and Aherne, 1980; Eisenhauer et al., 1994). The rather low K concentrations in our study (3.2 ± 0.7 mmol/l) suggest that the phase of increased newborn hemolysis yielding higher K serum concentrations is almost completed at the time of baseline measurements. GOT and creatinine in 18 three days old piglets were 36 ± 6 U/l and 0.47 ± 0.03 mg/dl at baseline in a cecal ligation model (Goto et al., 2012). Data on urine production dependent on body weight have not yet been published to the best of our knowledge. Urine production depends on fluid intake and post-natal age and averages in the human infant between 2 and 5 ml/kg/h. The fluid intake in our protocol followed accepted guidelines (Petersen et al., 2003) and consisted of ~200 ml/kg/d consisting of ¾ enteral nutrition fluids and ¼ intravenous fluids.

**Table 2:** Electrolytes, renal function, and GOT.

		**Baseline**	**24 h**	**48 h**	**72 h**	**Sphericity**	**Time**	Time*group	**Group**
Sodium (mmol/l)	Total	141 ± 2	141 ± 2	143 ± 4	144 ± 4	0.00	0.00	0.86	0.94
	Control	142 ± 2	141 ± 2	143 ± 2	144 ± 2				
	Treat	141 ± 2	141 ± 3	143 ± 4	145 ± 4				
Potassium (mmol/l)	Total	3.2 ± 0.7	4.5 ± 0.7	3.9 ± 0.4	3.8 ± 0.3	0.00	0.00	0.30	0.22
	Control	2.8 ± 0.8	4.7 ± 0.6	3.7 ± 0.1	3.7 ± 0.2				
	Treat	3.3 ± 0.7	4.5 ± 0.7	3.9 ± 0.5	3.8 ± 0.3				
Calcium (mmol/l)	Total	2.6 ± 0.1	2.4 ± 0.1	2.5 ± 0.1	2.5 ± 0.1	0.10	0.00	0.29	0.98
	Control	2.6 ± 0.1	2.5 ± 0.1	2.5 ± 0.1	2.5 ± 0.1				
	Treat	2.6 ± 0.1	2.4 ± 0.1	2.5 ± 0.1	2.5 ± 0.1				
Chloride (mmol/l)	Total	103 ± 3	106 ± 3	106 ± 4	107 ± 5	0.00	0.00	0.62	0.76
	Control	104 ± 3	107 ± 3	106 ± 3	108 ± 5				
	Treat	103 ± 3	106 ± 3	107 ± 4	107 ± 5				
Creatinine (mg/dl)	Total	0.53 ± 0.09	0.60 ± 0.21	0.51 ± 0.12	0.42 ± 0.08	0.00	0.00	0.69	0.26
	Control	0.54 ± 0.08	0.66 ± 0.16	0.53 ± 0.12	0.45 ± 0.07				
	Treat	0.53 ± 0.09	0.59 ± 0.22	0.50 ± 0.13	0.41 ± 0.08				
Urine output (ml/kg/h)					2.2 ± 0.8				
GOT (AST) (IU/l)	Total	31 ± 6	51 ± 27	53 ± 37	39 ± 32	0.00	0.00	0.85	0.74
	Control	31 ± 6	46 ± 26	54 ± 45	35 ± 28				
	Treat	31 ± 7	52 ± 27	52 ± 35	40 ± 33				

*GOT (AST), glutamic oxaloacetic aminotransferase (aspartate aminotransferase)*.

*Data are mean ± SD. Number of piglets included: total = 51, control = 8, treat(ment) = 43. Comparisons by two-way mixed ANOVA for repeated measures data*.

### Blood Cell Differentials

We observed time-dependent changes in all blood cell lines (monocytes excepted) and a significant interaction for thrombocytes (time^*^group). Most of the cell lines did not show a clear trend, the administration of LPS at 48 h included (Table 3). The hematocrit of 2–5 days old piglets was 27 ± 2% (equivalent to a hemoglobin concentration of 9.0 ± 0.6 g/dl) (Eisenhauer et al., 1994) and 8.5 ± 3.2 g/dl in piglets on day 1 and 2 (Park and Chang, 2000).

**Table 3:** Blood cell differentials.

		**Baseline**	**24 h**	**48 h**	**72 h**	**Sphericity**	**Time**	Time*group	**Group**
Hemoglobin (g/dl)	Total	8.0 ± 1.1	7.8 ± 1.1	7.5 ± 0.9	7.5 ± 0.8	0.00	0.00	0.31	0.53
	Control	8.2 ± 0.8	8.2 ± 0.9	7.5 ± 0.6	7.6 ± 0.8				
	Treat	7.9 ± 1.2	7.7 ± 1.1	7.5 ± 0.9	7.5 ± 0.8				
Leukocytes (cells/nl)	Total	15.0 ± 6.6	21.3 ± 8.7	15.5 ± 6.6	12.4 ± 5.7	0.20	0.00	0.31	0.06
	Control	16.1 ± 9.2	26.3 ± 13.2	18.4 ± 10.4	15.2 ± 7.0				
	Treat	14.8 ± 5.9	20.1 ± 7.1	14.8 ± 5.3	11.8 ± 5.2				
Thrombocytes (cells/nl)	Total	443 ± 97	459 ± 92	414 ± 132	396 ± 143	0.00	0.00	0.01	0.69
	Control	494 ± 141	464 ± 109	372 ± 160	340 ± 161				
	Treat	431 ± 82	457 ± 89	424 ± 124	410 ± 138				
Bands (%)	Total	12 ± 8	12 ± 9	7 ± 6	13 ± 8	0.15	0.00	0.31	0.67
	Control	12 ± 7	16 ± 15	5 ± 7	15 ± 10				
	Treat	12 ± 8	11 ± 6	7 ± 5	13 ± 8				
PMNL (%)	Total	68 ± 10	72 ± 11	69 ± 11	57 ± 14	0.07	0.00	0.42	0.70
	Control	68 ± 10	69 ± 16	72 ± 11	61 ± 14				
	Treat	67 ± 10	72 ± 10	68 ± 12	56 ± 14				
Lymphocytes (%)	Total	16 ± 7	13 ± 6	20 ± 11	25 ± 11	0.00	0.00	0.62	0.55
	Control	17 ± 9	12 ± 5	20 ± 12	21 ± 10				
	Treat	16 ± 6	14 ± 6	21 ± 10	26 ± 11				
Monocytes (%)	Total	2 ± 1	1 ± 1	1 ± 2	1 ± 2	0.13	0.67	0.34	0.62
	Control	1 ± 1	1 ± 1	1 ± 1	2 ± 2				
	Treat	2 ± 1	1 ± 1	1 ± 2	1 ± 2				

*PMNL, polymorphonuclear leukocytes*.

*Data are mean ± SD. Number of piglets included: total = 51, control = 8, treat(ment) = 43. Comparisons by two-way mixed ANOVA for repeated measures data*.

Clearly, the hematocrit of term newborns at 48 h of age is higher [17.7 g/dl ± 1.8 to 19.5 ± 2.1 depending on the mode of cord clamping (Mercer et al., 2017)] thus doubling the oxygen transport capacity and making the human newborn less vulnerable to an impaired gas exchange in the transitional period.

### Lung Function

The determination of EVLWI has been performed by the thermodilution method in newborn piglets yielding a value of 20 ± 1 ml/kg (Silvera et al., 2011) and in human neonates following arterial switch operation due to transposition of the great arteries yielding 20 ± 7 ml/kg after extubation (Székely et al., 2011), however data in well babies do not exist because of the invasiveness of the technique. In (adult) humans a value of 3–7 ml/kg is considered normal, however neonates tend to have higher values because of incomplete resorption of lung fluids in the post-natal transitional process and of shunting via a patent ductus arteriosus and foramen ovale. Our baseline data of “total” (13.2 ± 5.5 ml/kg, Table 4) are close to the values of newborn neonatal lambs assessed by multiple indicator dilution methods showing an EVLWI of 10.7 ± 1.4 ml/kg (Sundell et al., 1987).

**Table 4:** Lung function.

		**Baseline**	**24 h**	**48 h**	**72 h**	**Sphericity**	**Time**	Time*group	**Group**
EVLWI (ml/kg)	Total	13.2 ± 5.5	18.1 ± 9.4	18.4 ± 7.9	23.3 ± 8.4	0.01	0.00	0.16	0.00
	Control	15.5 ± 5.4	25.0 ± 5.5	25.0 ± 5.3	30.6 ± 5.7				
	Treat	12.7 ± 5.5	16.8 ± 9.5	17.2 ± 7.7	22.0 ± 8.1				
C_rs_ (ml/mbar/kg)	Total	1.14 ± 0.51	0.72 ± 0.30	0.60 ± 0.23	0.57 ± 0.19	0.00	0.00	0.50	0.22
	Control	1.14 ± 0.40	0.69 ± 0.25	0.51 ± 0.09	0.42 ± 0.12				
	Treat	1.14 ± 0.53	0.73 ± 0.31	0.62 ± 0.25	0.61 ± 0.19				
R_rs_ (mbar/l*s)	Total	59 ± 11	81 ± 30	98 ± 40	97 ± 36	0.00	0.00	0.00	0.00
	Control	57 ± 13	80 ± 25	125 ± 55	141 ± 57				
	Treat	60 ± 10	82 ± 31	92 ± 33	87 ± 20				
FRC (ml/kg)	Total	28.7 ± 6.0	19.5 ± 6.2	–	–	–	0.00	0.04	0.18
	Control	28.7 ± 6.3	18.4 ± 5.4						
	Treat	28.7 ± 6.3	23.8 ± 7.5						
V_A_ (ml/kg)	Total	2.3 ± 0.9	2.0 ± 0.9	–	–	–	0.08	0.20	0.12
	Control	2.1 ± 0.8	1.4 ± 0.8						
	Treat	2.4 ± 0.9	2.3 ± 0.9						
OI (MAP*%O_2_/PaO_2_)	Total	2.3 ± 0.7	5.8 ± 3.4	7.2 ± 3.8	8.7 ± 4.9	0.03	0.00	0.00	0.14
	Control	2.0 ± 0.6	5.0 ± 2.7	6.9 ± 3.0	16.1 ± 6.1				
	Treat	2.4 ± 0.6	6.0 ± 3.5	7.2 ± 4.0	7.5 ± 3.7				
VEI (3,800/(PIP-PEEP*f*PaCO_2_))	Total	0.38 ± 0.19	0.17 ± 0.09	0.19 ± 0.10	0.15 ± 0.07	0.00	0.00	0.00	0.13
	Control	0.53 ± 0.23	0.18 ± 0.09	0.19 ± 0.08	0.14 ± 0.09				
	Treat	0.35 ± 0.17	0.17 ± 0.09	0.19 ± 0.11	0.15 ± 0.06				

*EVLWI, extra-vascular lung water index; sC_rs_, specific compliance of the respiratory system; R_rs_, resistance of the respiratory system; FRC, functional residual capacity; V_A_, alveolar ventilation; OI, oxygenation index; VEI, ventilation efficiency index*.

*Data are mean ± SD. Number of piglets included: total = 51, control = 8, treat = 43 (FRC/V_A_: total = 22, control = 7, treat = 15). Comparisons by two-way mixed ANOVA for repeated measures data*.

Baseline sC_rs_ in mechanically ventilated neonatal piglets has been determined by many researchers with values of 1.34 ± 0.11 ml/mbar/kg (Sood et al., 1996a), 0.79 ± 0.15 (Khan et al., 1999), 1.5 ± 0.3 (Tølløfsrud et al., 2002), 0.95 ± 0.05 (Dargaville et al., 2003), 1.03 ± 0.33 (Meister et al., 2004), 1.38 ± 0.15 (Chada et al., 2008), and 1.07 ± 0.17 (Yang et al., 2010). These data are close to our “total” value of 1.14 ± 0.51 ml/mbar/kg, in contrast to R_rs_ values (“total”: 59 ± 11 mbar/l^*^s) showing greater variations which occur due to differences in body weight, endotracheal tube size and leakage, amount of continuous flow in the ventilator tubings, and medication: 32 ± 3 mbar/l^*^s (Sood et al., 1996a), 88 ± 9 (Tølløfsrud et al., 2002), 74 ± 4 (Dargaville et al., 2003), and 64 ± 8 (Meister et al., 2004). FRC is considered the main determinant of oxygenation and is deemed to be significantly reduced by any lung injury protocol (“control”: from 28.7 ± 6.3 ml/kg to 18.4 ± 5.4 after 24 h of mechanical ventilation following repeated airway lavage); FRC was 21.8 ± 2.4 ml/kg in three days old piglets (Standaert et al., 1991) and 21 ± 2 in 5–7 days old piglets (Meister et al., 2004). To the best of our knowledge, data on V_A_ have not been published by other investigators but were measured with 4.8 ± 0.3 ml/kg in a previous study of our group (Krause et al., 2001).

Impairment of oxygenation is a prerequisite of P/NARDS and is usually defined by the OI which is an equation composed of the degree of respiratory support (mean airway pressure, MAP), the oxygen concentration in respiratory gas mixtures, and the partial pressure of O_2_ in blood as a measure of gas exchange (MAP^*^%O_2_/PaO_2_). By the Montreux definition of NARDS (De Luca et al., 2017), the control group experienced severe NARDS expressed by an OI of 16.1 ± 6.1 at 72 h of mechanical ventilation (Table 4). Baseline values in our study (“total”: 2.3 ± 0.7) are close to those from other investigators: 1.5 ± 0.5 ml/mbar/kg (Khan et al., 1999), 1.4 ± 0.3 (Tølløfsrud et al., 2002), and 1.3 ± 0.3 (Renesme et al., 2013). The VEI in “total” (0.38 ± 0.19) is close to the value of 6 days old piglets at baseline (0.30 ± 0.02) in the lavage study by Sood et al. (1996a) and to the value of 5 days old piglets (0.33 ± 0.08) in the meconium aspiration study by Khan et al. (1999).

### Bacteria in Airways

A plentitude of different bacteria in the airways was cultured with the initial lavage mainly belonging to the three groups of (lacto)bacillales, enterobacteriaceae, and soil-based bacteria (Table 5). Given the relative dominance of soil-based bacteria in the airways of our piglets (Bacillus cereus, Rothia, aerobic spore builder, Corynebacterium sp.) inhalation of these microorganisms due to the use of the piglets' nose for foraging and consecutive colonization of upper and lower airways must be considered. The high frequency in bacillus cereus colonization (in 8/52 cultures from the final lavages) demonstrates the natural resistance to beta-lactams, e.g., ampicillin ± sulbactam as given in our study (Glasset et al., 2018). The increasing prevalence of colonization by multidrug resistant Gram-negative bacteria, such as *E. coli* and *Klebsiella* sp. in neonatal intensive care units (NICU) are correlated with length of NICU stay, and—indeed—exposure to ampicillin/sulbactam (Giuffrè et al., 2016).

**Table 5:** Bacteria in airways.

		**Initial lavage (0 h)**			**Final lavage (72 h)**		
		**+**	**++**	**+++**	**+**	**++**	**+++**
(Lacto)bacillales	*Staphylococcus aureus*	2	1	0			
	*Staphylococcus hemolyticus*	1	0	0			
	*S. aureus*				0	0	1
	*Streptococcus*	8	4	0			
	*Streptococcus suis*	3	6	0			
	*Enterococcus*	5	0	1	1	1	1
Enterobacteriaceae	*Escherichia coli*	6	2	0	5	16	11
	*Proteus* sp.	1	0	0	0	1	0
	*Klebsiella*				1	1	5
	*Pasteurella*	0	1	0			
	*Oligella*	1	0	0			
	*Moraxella*	1	1	0			
Diverse	*Pharyngeal flora*	6	3	0			
Soil-based bacteria	*Bacillus cereus*	1	0	0	1	3	4
	*Rothia*	1	1	0			
	Aerobic spore builder	2	0	0	3	4	1
	*Corynebacterium* sp.	0	1	0			

*N = 51 at both times. Bacterial growth from BALF on Agar plates: single colonies on plate (+), sparse growth (++), intermediate growth (+++). Piglets with heavy growth were excluded from the final data analysis [n = 3; E. coli (2), Klebsiella]. More than one bacterium was grown in some of the BALFs. All piglets received ampicillin/sulbactam at a dosage of 100 mg/kg twice a day. Overgrowth of E. coli, Klebsiella, Bacillus cereus, and aerobic spore builders due to natural or acquired resistance*.

### Lung and Body Weights

We determined a lung/body weight relation of 1.6 ± 0.2% (Table 6) which is in line with the findings in 6 three days old piglets [1.5 ± 0.2 (Standaert et al., 1991)], of 1.0 ± 0.1 in 8 fourteen days old piglets (Dargaville et al., 2003), 3.0 ± 0.3 in 13 three days old piglets (van Kaam et al., 2004b), and 1.7 ± 0.1 in 27 one day old piglets (Miles et al., 2012). The applicability of the neonatal piglet lung model for studying severe lung diseases is also expressed by the similarities to term human lung/body relations of 1.7 ± 0.4% (De Paepe et al., 2005) and 1.9 ± 0.3 (De Paepe et al., 2014).

**Table 6:** Lung and body weights.

	**HC_**0**_**	**C_**72**_**	**T_**72**_**	**HC_**0**_ vs. C_**72**_**	**C_**72**_ vs. T_**72**_**
Lung weight (g)	40 ± 4	75 ± 8	69 ± 9	<0.0001	0.13
Body weight (kg)	2.4 ± 0.2	2.3 ± 0.1	2.3 ± 0.1	0.29	0.38
Body weight gain (kg)	–	0.15 ± 0.04	0.11 ± 0.05	–	0.05
Lung/body weight (%)	1.6 ± 0.2	3.2 ± 0.4	2.9 ± 0.4	<0.0001	0.07

*HC_0_, healthy controls at 0 h; C_72_, controls at 72 h; T_72_, treated at 72 h; body weight gain, weight gain within 72 h of mechanical ventilation including triple-hit lung injury*.

*Data are mean ± SD. Number of piglets included: HC_0_ = 8, control = 8, treat(ment) = 43. Comparison by t-tests*.

### Cells in BALF and Apoptosis

There is currently no reliable indicator to assess the amount of epithelial lining fluid recovered by broncho-alveolar lavage (de Blic et al., 2000). Most commonly urea and albumen have been used as reference substances, however, lower serum concentrations of both substances in the smallest children bedevil the interpretation of cellular and non-cellular concentrations in BALF, as do the size of the lungs, the region of interest within the lung (in the context of bronchoscopic BALF recovery), the amount of lavage fluid used, the aspiration technique, and the processing of cellular and non-cellular components. The lavage protocol used in our studies consisted of the instillation and aspiration of 30 ml/kg of warmed normal saline by a syringe hooked up to the adaptor of the endotracheal tube.

**Figure 1:**
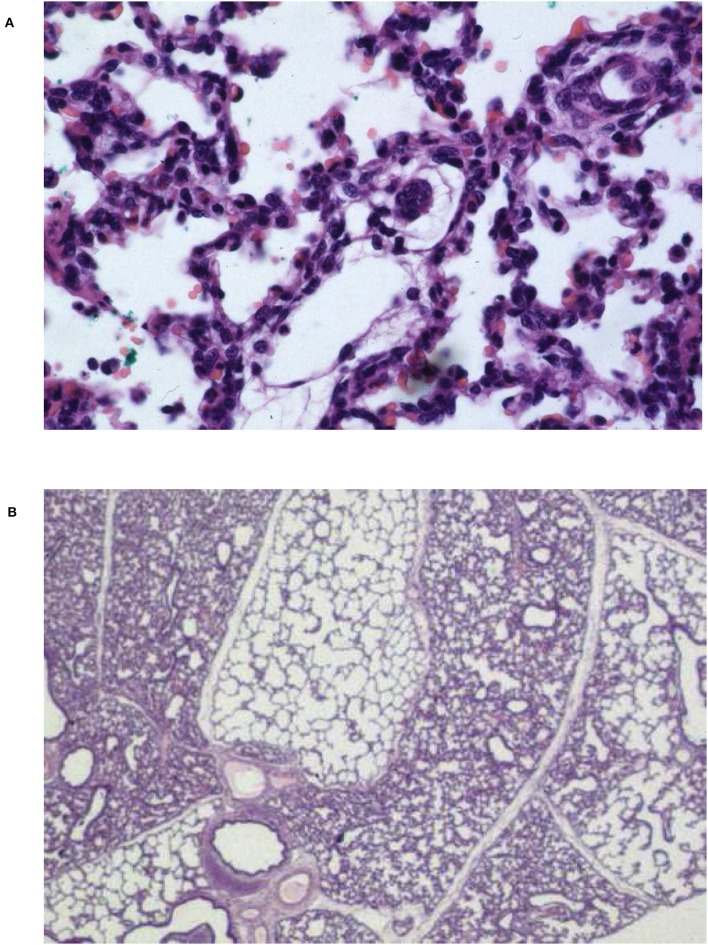
Microscopic findings of a control piglet after 24 h of mechanical ventilation following repeated airway lavage. **(A)** Diffuse alveolar collapse next to overdistention, severely thickened alveolar septae. Some hyaline membrane formation and alveolar basement denudation is evident. Abundant cellular infiltration with PMNL and macrophages in the pulmonary interstitium and in the alveolar spaces. Infiltrations with red blood cells as sign of diffuse pulmonary hemorrhage and coagulation activation. **(B)** Lobuli with alveolar collapse adjacent to overdistention containing proteinaceous alveolar edema. Hematoxylin and eosin staining, oil × 1,200 **(A)**, ×300 **(B)**.

An increased BALF total cell count >150 cells/μl is a common characteristic of many lung diseases in infants and children (Riedler et al., 1995). Thus, the total cell count of 633 ± 336/μl (Table 7) in our study at baseline suggests an important impact of bacterial colonization in the majority of the piglets (43/51 = 84%). The dominance of alveolar macrophages in newborns/young infants with ~98% in cell differentials changes over time and reaches ~90% at an age of 7 years (Grigg and Riedler, 2000), linked with an appropriate increase of the lymphocyte counts. Not surprisingly the PMNL count of 32 ± 14% in our study is much higher than in human newborns. Following meconium instillation in one lung lobe and mechanical ventilation of 12 h the total cell count was 1,400 ± 1,100/μl in 17 piglets at day 0–2 of life (Korhonen et al., 2004). Likewise PMNL was the dominating cell line (1,000 ± 900/ml) as also seen in our model (80 ± 4%) (Figure 1).

**Table 7:** Cells in BALF and apoptosis.

	**Total_**0**_**	**C_**72**_**	**T_**72**_**	**Total_**0**_ vs. C_**72**_**	**C_**72**_ vs. T_**72**_**
Total cells (cells/μl)	633 ± 336	1,624 ± 1,003	1,059 ± 786	<0.0001	0.07
PMNL (%)	32 ± 14	80 ± 4	79 ± 7	<0.0001	0.95
Lymphocytes (%)	3.2 ± 3.2	1.9 ± 1.0	1.9 ± 0.9	0.13	0.97
Monocytes/macrophages (%)	64 ± 15	17 ± 4	18 ± 7	<0.0001	0.89
CD14^+^/18^+^ (%)	28 ± 15	64 ± 15	59 ± 25	0.003	0.94
Apoptotic PMNL (%)	10.2 ± 8.3	16.2 ± 6.6	11.2 ± 5.9	0.04	0.08
Apoptotic macrophages (%)	10.5 ± 11.1	54.8 ± 24.6	48.3 ± 20.8	0.0004	0.44
Apoptotic AEC (%)	15.0 ± 5.8	15.0 ± 5.6	13.9 ± 5.7	1.00	0.68

*BALF, broncho-alveolar lavage fluid; PMNL, polymorpho-nuclear leukocytes; AEC, alveolar epithelial cells*.

*Total_0_, all piglets at 0 h; C_72_, controls at 72 h; T_72_, treated piglets at 72 h*.

*Data are mean ± SD. Number of piglets included: Total_0_ = 51, control = 8, treat(ment) = 43. Comparison by t-test for total cells, and by Mann-Whitney tests for all other parameters*.

PMNL, monocytes, and lung macrophages express CD14 implicated in the cellular response to LPS (given intratracheally as part of the triple-hit lung injury protocol applied here) together with a plasma LPS-binding protein. MD-2 and the intracellular part of TLR4 are necessary for the transduction of the signal activating cytokine and chemokine genes. The β2-integrin CD18 is also expressed by both, PMNL and monocytes/macrophages, and plays an important role in the migration of cells to areas of the lung containing high concentrations of chemokines, such as C5a. Monocytes recruited into the alveolar space keep phenotypic features of blood monocytes but upregulate CD14 resulting in enhanced responsiveness to LPS with increased cytokine expression (Maus et al., 2001). 28 ± 15% of the cells harvested by BALF (Table 7) are CD14^+^/18^+^ and belong to either population; their response to LPS and the concomitant (overwhelming) production of TNF-α, IL-1α and IL-1β, IL-6, IL-8, C3a, and C5a (Billman Thorgersen et al., 2009) represents a major pro-inflammatory pathway in the ARDS lung (Dentener et al., 1993). An important difference in physiologic response of the porcine lung to a variety of agents, such as particulates, bacteria, fibrin, cellular debris, and immune cells are constitutive pulmonary intravascular macrophages (PIM) that express a β3 integrin subunit (CD61) for the clearance of all kinds of proteins from the circulation (Schneberger et al., 2012). The heavy accumulation of PIM in lung tissue is linked with an increase in vascular permeability, edema, hemorrhage, and alveolar septal thickening (Figure 1) in a piglet model of classical swine fever (Núnez et al., 2018).

Alveolar epithelial apoptosis is a typical feature of the ARDS lung (15.0 ± 5.8%, Table 7) and is linked with impairment of oxygenation and ventilation and abrogated barrier functions (Matute-Bello and Martin, 2003). In pediatric patients dying from PARDS the extent of cleaved caspase-3 in alveolar epithelial cells as a surrogate parameter of apoptosis has been quantified by Bern et al. (2010) yielding a percentage of 6.4 ± 1.2 (range 1.0–18.1)%. Apoptosis in severe lung disease must be differentiated from apoptosis during the process of alveolarization and differentiation which continues after birth until the second year of life; thus background apoptosis of 1–2% of AEC must be considered in neonatal organisms when evaluating lung disease (del Riccio et al., 2004). In ARDS the percentage of apoptotic PMNL obtained by lavage was 3 (0–7.3)% in patients who died (Matute-Bello et al., 1997), and 10–20% in a murine ARDS model of intraperitoneal LPS (data on human or porcine neonates unknown) (Wang et al., 2014). Data on macrophage apoptosis are scarce and increase from 10.1 ± 1.1% to 20.2 ± 1.7 following LPS challenge in murine cell cultures (Li et al., 2018).

### Surfactant Surface Tension and Alveolo-Capillary Leakage

Regardless of the kind of acute lung injury the surfactant surface tension (Table 8) will increase considerably due to either a loss of the surfactant pool (repeated airway lavage) or disturbances in surfactant function (meconium instillation, LPS instillation, hyperoxia). In a meconium aspiration model minimum surfactant surface tension increased from 17.8 ± 4.8 mN/m to 23.3 ± 4.8 (Wiswell et al., 1994), and in a repeated airway lavage model from 11.1 ± 5.2 to 21.8 ± 2.1 (von Bismarck et al., 2007). Albumen has been identified as a major factor of surfactant inhibition (Seeger et al., 1993) and simultaneously reflects the degree of capillary-alveolar leakage as part of the inflammation of lung tissue and pulmonary capillaries. Albumen concentrations in BALF have been assessed in a hyperoxia model with baseline values of 56 ± 19 μg/ml and a 3-fold increase following lung injury (Davis et al., 1989). SP-D serum concentrations in ARDS increased 3- to 4-fold [from 1.9 μg/ml (0.6–4.4) to 5.9 (2.5–22.7) (Yang et al., 2017); and from 83 ± 33 ng/ml to 476 ± 391 (Endo et al., 2002)]. SPLA_2_ has been blamed to play a major role in surfactant degradation in NARDS lungs (De Luca et al., 2017) as evidenced in 10 neonates with severe sepsis/pneumonia [control: 0.5 IU/ml (0.1–3.1), nARDS: 4.0 (2.1–8.5)] (De Luca et al., 2008). Not surprisingly, sPLA_2_ also plays an important role in PARDS (infants between 2 and 10 months of age) with activities being increased by factor three compared to control groups and with significant correlation of sPLA_2_ changes and changes in free fatty acid concentrations in BALF; in addition, TNF-α concentrations, surfactant phospholipids, and surface tension from epithelial lining fluid were correlated to sPLA_2_ variations (De Luca et al., 2011, 2013). BALF SP-A levels remained almost constant in a piglet model of repeated airway lavage and the installation of group B streptococci into the airways [healthy: 80 ± 43 pg/ml, treat 74 ± 34 (van Kaam et al., 2004b)], as there were no significant differences in SP-A gene expression in alveolar epithelial cells between aARDS and control patients (Pires-Neto et al., 2013).

**Table 8:** Surfactant surface tension and alveolar-capillary leakage.

	**Total_**0**_**	**C_**72**_**	**T_**72**_**	**Total_**0**_ vs. C_**72**_**	**C_**72**_ vs. T_**72**_**
Surfactant surface tension (m/Nm)	21 ± 2	41 ± 14	28 ± 8	0.0004	0.001
sPLA_2_ in BALF (ng/ml)	20 ± 10	28 ± 11	14 ± 10	0.06	0.006
Albumen in BALF (μg/ml)	111 ± 25	378 ± 36	275 ± 115	<0.0001	0.015
SP-A in BALF (%)	100 ± 3	68 ± 35	82 ± 35	<0.0001	0.285
SP-D in serum (ng/ml)	56 ± 3	326 ± 19	220 ± 101	<0.0001	0.005

*Surfactant surface tension, minimum surface tension of 10 mg surfactant in 1 ml BALF measured by a Wilhelmy balance; sPLA_2_, secretory phospholipase A_2_; SP-D, surfactant protein D*.

*Data are mean ± SD. Total_0_, all piglets at 0 h; C_72_, controls at 72 h; T_72_, treated piglets at 72 h. Number of piglets included: total_0_ = 51, control = 8, treat(ment) = 43. Comparisons by t-tests (SP-A by Mann-Whitney test)*.

### NF-κB, Inflammasome, and Ceramide Pathway

In an experimental pneumonia model with *E. coli* instilled into the airways of 3–4 weeks old piglets the NF-κB concentration in lung tissue homogenates increased from 0.25 to 0.4 arbitrary units and could be reduced by the application of inhaled nitric oxide or the instillation of surfactant (Zhu et al., 2005). The selective topical inhibition of NF-κB by IKK-NBD peptide via instillation into the airways using surfactant as a carrier substance improved FRC, V_A_, C_rs_, R_rs_, and EVLWI in a newborn piglet lavage model (Ankermann et al., 2005a; von Bismarck et al., 2007). Of note, the reduction of NF-κB activity in the nucleus of pulmonary cells from 100 ± 2% to 32 ± 2 by IKK-NBD peptide was more pronounced than the effect of dexamethasone reaching an activity of only 55 ± 4% (von Bismarck et al., 2009).

In porcine alveolar macrophages swine influenza virus induces massive IL-1β production secondary to an increased expression of inflammasome components (NLRP3, ASC, procaspase-1) (Park et al., 2018). In C57BL/6 mice the application of a two-hit lung injury by mechanical ventilation and LPS induces IL-1β and KC (a murine functional analog of IL-8), and cell migration into the alveolar space, all of which may be considerably reduced by the administration of the IL-1 antibody anakinra (Jones et al., 2014). NLRP^−/−^ mice exposed to hyperoxia showed significantly lower IL-1β, TNF-α, and MIP-2 concentrations in BALF (Fukumoto et al., 2013). The mutual dependency of the ceramide pathway and the inflammasome NLRP3 has been shown by Kolliputi and our group in alveolar epithelial cells (Kolliputi et al., 2012) and in porcine lung homogenates (Spengler et al., 2018) (Table 9). In tracheal aspirates from preterm infants prone to bronchopulmonary dysplasia (BPD) high IL-1β and IL-1ra concentrations were linked with more severe grades of BPD or death (Liao et al., 2018). In adult patients subject to overventilation (V_T_ = 12 ml/kg) ASC-upregulation in alveolar epithelial cells was ~10-fold compared to normoventilation, the expression of NLRP3 and ASC in alveolar macrophages doubled (Kuipers et al., 2012). The NLRP-dependent cytokine IL-1β was treated with either aerosolized or intravenous anakinra in a lavage model of surfactant depletion yielding moderately improved oxygenation, ventilation, C_rs_, and neutrophil migration into lung tissue (Chada et al., 2008). The IL-1β/β-actin ratio in lung tissue was reduced from 4.9 ± 2.4 (control) to 0.9 ± 0.3 (aerosolized) and 0.8 ± 0.1 (intravenous), respectively. A comparable reduction in the IL-8/β-actin ratio could be demonstrated. In an E. coli LPS model of ARDS treating 4–6 weeks old piglets by the intravenous route, IL-1β concentrations rose from 29 ± 2 pg/ml to 89 ± 18, IL-6 from 18 ± 8 pg/ml to 22 ± 7, and IL-8 from 80 ± 7 pg/ml to 118 ± 10 (Wang et al., 2016).

**Table 9:** NF-κB, inflammasome, and ceramide pathways.

	**HC_**0**_**	**C_**72**_**	**T_**72**_**	**Total_**0**_ vs. C_**72**_**	**C_**72**_ vs. T_**72**_**
Iκ-Bα (%)	96 ± 4	8 ± 4	35 ± 22	0.0002	0.0002
IκBkinase (%)	23 ± 9	99 ± 4	55 ± 27	0.0002	<0.0001
NF-κB (aU)		1.00 ± 0.28	0.69 ± 0.24		0.0012
NLRP3 (%)	29 ± 6	100 ± 7	77 ± 23	0.0022	0.0037
ASC (%)	27 ± 7	99 ± 7	72 ± 28	0.0022	0.0096
Cathepsin D (%)	33 ± 9	100 ± 7	74 ± 24	0.0002	0.0007
Caspase-1 (%)	27 ± 7	100 ± 5	81 ± 29	0.0002	0.0581
IL-1β (%)	31 ± 14	98 ± 11	77 ± 29	0.0002	0.0659
IL-18 (%)	27 ± 8	100 ± 12	83 ± 28	0.0012	0.1159
aSMase activity lung (nmol/mg/h)	18 ± 2	49 ± 4	35 ± 9	<0.0001	0.0001
Ceramide C16/18 lung (pmol)	537 ± 93	1,294 ± 189	987 ± 216	<0.0001	0.0005
aSMase activity liver (nmol/mg/h)	15 ± 4	53 ± 4	41 ± 11	<0.0001	0.0059
Ceramide C16/18 liver (pmol)	628 ± 129	1,446 ± 165	1,224 ± 240	<0.0001	0.0166

*Iκ-Bα, inhibitor of NF-κB release and translocation; IκBkinase, kinase of Iκ-Bα; aU, arbitrary units for C24 and T24; NLRP3, inflammasome nucleotide-binding domain–leucine-rich repeat-containing protein-3; ASC, apoptosis-associated speck-like protein containing a caspase recruitment domain; aSMase, acid sphingomyelinase*.

*HC_0_, healthy controls at 0 h; C_72_, controls at 72 h; T_72_, treated piglets at 72 h*.

*Data are mean ± SD. Number of piglets included: total = 51, control = 8, treat = 43 (NF-κB: C_72_ = 7, T_72_ = 15). Comparisons by Mann-Whitney tests for NF-κB and inflammasome parameters, by unpaired t-tests for ceramide parameters*.

“Ceramide lances the lungs,” as pointed out by P. Barnes (Barnes, 2004) describes the impact of the activated ceramide pathway on impairment of alveolo-capillary barrier functions in lung inflammation (Göggel et al., 2004). More than 30 years ago high concentrations of galactosylceramide (20- to 40-fold normal) were found in the lavage fluid of mechanically ventilated ARDS patients (Rauvala and Hallman, 1984). In (adult) patients suffering from cystic fibrosis the application of amitriptyline normalizes pulmonary ceramide and improves lung function including susceptibility to infection (Teichgräber et al., 2008). As it is well-known for many years that the porcine organism displays all kinds of glycolipids, such as galactosylceramide, glucosylceramide, ganglioside, and globoside (Kyogashima et al., 1989) there is unfortunately no data for comparing our results with regard to the impact of the acid sphingomyelinase/ceramide pathway on lung function. For the rat it has been shown that sphingomyelin content, sphingosine concentrations, and ceramide concentrations are highest in neonatal compared to fetal or adult lungs (Longo et al., 1997) underlining the important role of the ceramide pathway in neonatal lung physiology. In the newborn rat (Husari et al., 2006) and newborn mice (Tibboel et al., 2013) hyperoxia models, ceramide and sphingomyelin concentrations are increased 2- to 4-fold. In addition stretch applied to alveolar epithelial cells from newborn rat lungs by mechanical ventilation induces autophagy, acid sphingomyelinase activity, and ceramide generation (Yeganeh et al., 2018).

### Pro-fibrotic and Pro-inflammatory Parameters

TNF-α in BALF (Table 10) increased from 21 ± 4 pg/ml to 42 ± 22 following meconium instillation into the lungs of 1–2 days old piglets (Korhonen et al., 2004), from 0.03 ± 0.02 U/ml to 0.34 ± 0.58 in a newborn piglet lavage model (Krause et al., 2005), and from 80 ± 84 pg/ml to 1,357 ± 676 in a meconium model with 1–3 days old piglets (Angert et al., 2007). Depending on the kind of acute lung injury the increasing pre-/post-injury factor varies largely between 1:2 and 1: 60 (Table 10). IL-8 concentrations rose from 51 ± 34 pg/ml to 429 ± 259 in a lavage model (Ankermann et al., 2005b), and from 406 ± 364 pg/ml to 4,837 ± 1,951 in an meconium aspiration model (Angert et al., 2007), whereas IL-6 from BALF came up from 0.4 ± 1.0 U/ml to 29 ± 28 following repeated airway lavage (Krause et al., 2005). LTB_4_ as an important chemokine in the inflamed lung and increased from 2.6 ± 1.9 pg/ml to 9.3 ± 7.8 in a newborn lavage model (Ankermann et al., 2005a).

**Table 10:** Pro-fibrotic and pro-inflammatory parameters.

	**Total_**0**_**	**C_**72**_**	**T_**72**_**	**Total_**0**_ vs. C_**72**_**	**C_**72**_ vs. T_**72**_**
TGF-β (%)	20 ± 15	103 ± 2	84 ± 31	0.0079	0.0747
IFN-γ (%)	26 ± 14	100 ± 13	76 ± 29	0.0022	0.0571
Elastin (%)	48 ± 7	101 ± 5	74 ± 21	0.0002	0.0004
MMP-1 (%)	45 ± 7	101 ± 6	68 ± 22	0.0002	0.0003
IL-8 in BALF (pg/ml)*		455 ± 320	90 ± 101		0.0018
IL-6 in BALF (pg/ml)*		56 ± 14	19 ± 3		<0.0001
LTB_4_ in BALF (pg/ml)*		95 ± 72	45 ± 21		0.0267
TNF-α in BALF (pg/ml)	2 ± 6	117 ± 119	70 ± 66	<0.0001	0.1425

*TGF-β, transforming growth factor-β; IFN-γ, interferon-γ; MMP-1, matrix metalloproteinase 1; LTB_4_, leukotriene B_4_*.

Data are mean ± SD. Number of piglets included: total_0_ = 51, C_72_ = 8, T_72_ = 43 (*parameters: C_24_ = 7, T_24_ = 15). Comparisons by Mann-Whitney tests, BALF parameters by unpaired t-tests.

* *Parameters measured at C_24_ and T_24_*.

Data from other authors on fibrosis in (newborn and adult) piglets subject to induced acute lung injury are missing probably due to the observation interval of at least 24–72 h before changes in pro-fibrotic parameters may be quantified as demonstrated in ARDS patients (Fahy et al., 2003; Fligiel et al., 2006). A 72 h model of clinical observation as presented here (Preuß et al., 2012b; Spengler et al., 2018) is expensive and requires detailed knowledge of neonatal physiology and intervention skills. However, as an exception, von der Hardt et al. presented TGF-β mRNA expression data in a piglet lavage model unfortunately not yielding an adequate control group (von der Hardt et al., 2002). The variation of TGF-β between the four intervention groups in this study was not surprisingly very small (1.30 ± 0.11 to 1.79 ± 0.20 relative units) suggesting an inadequate observation time of 8 h only. TGF-β1 and its isoforms is constitutively stored by mammalian cells, may be released upon integrin signaling, and induce alveolar epithelial cell differentiation into (myo)fibroblasts which avidly produce collagen and elastin as part of the intermediate fibrotic stage in ARDS. Next to TGF-β1 and IFN-γ signaling the matrix metalloproteinase MMP-1 (in contrast to MMP-2, MMP-8, and MMP-9) plays a distinct role in fibrosis as high concentrations in BALF may discriminate patients surviving or not surviving ARDS (Fligiel et al., 2006).

### Systematic Review

**Figure 2:**
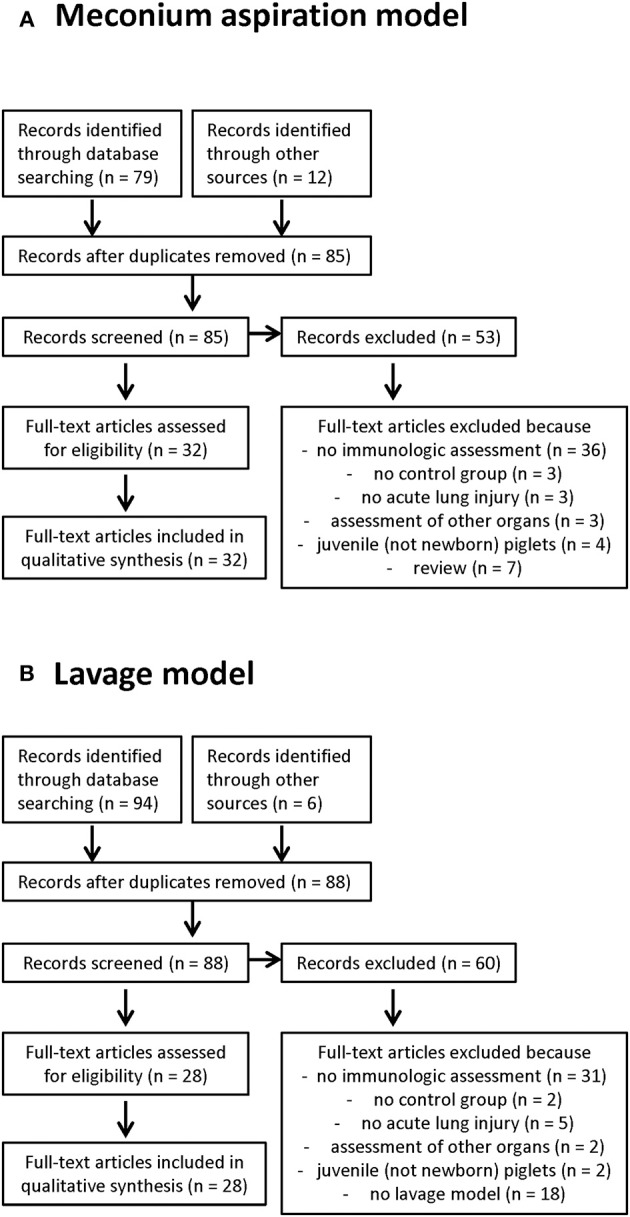
Flow sheets of the systematic review of the meconium aspiration model **(A)** and the lavage model **(B)**.

The systematic review (flowsheets in Figure 2) highlights two major acute direct lung injury models with the need of mechanical ventilation in term newborn piglets <14 days of age. Thus, gradually developing lung injury models, such as hyperoxia application or lung injury models without mechanical ventilation are not covered here. For a better understanding of NARDS immunologic outcome parameters and the effect of specific interventions are displayed.

### Meconium Aspiration Model

The meconium aspiration model is a frequently used model of direct lung injury by the installation of (human) diluted meconium into the airways. Within 2 h following meconium instillation, an increase in OI and R_rs_ and a decrease in sC_rs_ by ~50% can be observed (Kuo and Chen, 1999; Tølløfsrud et al., 2002). BP, CI and SVRI do not change significantly compared to control groups whereas the pulmonary arterial pressure (PAP) and the pulmonary vascular resistance index (PVRI) differ beyond a 2 h margin (Trindade et al., 1985; Kuo and Chen, 1999; Ryhammer et al., 2007). Of note, the deteriorations in lung mechanics and gas exchange are not sustained evaluating studies with longer observation periods (i.e., 12–48 h) when inflammatory parameters start to gradually decline again (Davey et al., 1993; Korhonen et al., 2004).

Meconium is composed of a myriad of substances essentially containing gastrointestinal secretions, bile, bile acids, pancreatic juice, mucus, swallowed vernix caseosa, lanugo hair, cellular debris, and blood (van Ierland and de Beaufort, 2009). As meconium is located “extracorporally” (i.e., hidden in the intestinal tract) its content normally is not recognized by the fetal immune system (Lindenskov et al., 2015). However, once meconium enters the airways the innate immunity senses a “damaged self” and reacts with “chemical pneumonitis” including increased airway responsiveness, pulmonary hypertension, cellular infiltration, impairment of gas exchange, PMNL infiltration of airways and lung tissue, alveolar epithelial cell apoptosis, and a cytokine storm (Lindenskov et al., 2005).

Therefore, aspects most often studied in the newborn piglet meconium aspiration model are cytokines/chemokines, PMNL infiltration (by the quantification of myeloperoxidase (MPO) in BALF and in lung tissue by immunohistochemistry), reactive oxygen species (ROS), pulmonary hypertension, arachidonic acid metabolites (notably sPLA_2_), and changes of the complement system (membrane attack protein sC5b-9).

Many studies quantified cytokines/chemokines, such as IL-1β, IL-6, IL-8, and TNF-α (Table 11) all of which largely depend on pattern recognition by the Toll-like receptor family (especially TLR4/MD-2, CD14, and C5a) (Salvesen et al., 2010). Specific therapy assessment to influence e.g., CD14 (Thomas et al., 2018) and downstream NF-κB by broad-acting glucocorticoids (Holopainen et al., 2001; Lin et al., 2016, 2017) or more specific inhibitors of NF-κB are scarce and deserve further evaluation.

**Table 11:** Meconium aspiration model.

**References**	**Age (days)**	**Meconium (%)**	**Volume (ml/kg)**	PIP/PEEP^*^ (mbar)	**${\text{V}}_{\text{T}}^{\#}$ (ml/kg)**	**Study l.^**$**^ (h)**	C_**rs**_/${\text{R}}_{\text{rs}}^{§}$ (ml/mbar/kg) (mbar/l^*^sec)	**PAP/PVR(I)^**¶**^ (mmHg) (mmHg/ml/kg/min a.o.^†^)**	**Intervention**	**Immunologic response**
Davey et al. (1993)	1–2	20	3	13/3	11 ± 1	48	1.5 ± 0.2 → 1.6 ± 0.4	–	None	Albumin: 20 ± 5 → 105 ± 35 μg/ml Protein: 1.1 ± 0.3 → 2.2 ± 0.3 mg/ml
							41 ± 5 → 162 ± 27	–		
Wiswell et al. (1994)	1–5	33	3	15/4 → 23/5	–	6	–	–	Beractant, poractatant	Protein: 1.1 ± 0.4 vs. 1.1 ± 0.2 mg/ml
							–	–		Phospholipids: 3.8 ± 1.5 vs. 6.2 ± 3.0 μg/ml
Holopainen et al. (1999a)	10–12	2, 6.5	3	/2–3	16–20	6	–	–	None	MPO: 9 ± 1 vs. 53 ± 13 U/g protein
										PLA_2_: 0.02 ± 0.01 vs. 0.16 ± 0.03 U/g protein
										AEC apoptosis: 7 ± 2 vs. 16 ± 2 cells/mm^2^
Holopainen et al. (1999b)	10–12	6.5	3	/2–3	16–20	6	–	–	NO 1, 10 ppm	MPO: 53 ± 13 vs. 57 ± 11 mU/mg protein
							–	11 ± 1 → 28 ± 2 mmHg/l/min		PLA_2_: 0.16 ± 0.03 vs. 0.11 ± 0.04 U/g AEC apoptosis: 16 ± 2 vs. 6 ± 3 cells/mm^2^
Kuo and Chen (1999)	<8	20	3	12 → 29/4	–	4	1.2 ± 0.4 → 0.3 ± 0.1	19 ± 3 → 31 ± 4	None	Blood endothelin-1: 1.6 ± 0.2 vs. 2.2 ± 0.4 pg/ml
							23 ± 5 → 35 ± 9	960 ± 290 → 2,620 ± 490		
Holopainen et al. (2001)	10–12	6.5	3	/2–3	16–20	6	–	13 ± 3 → 26 ± 7	Dexamethasone 0.5 mg	PLA_2_: 0.16 ± 0.07 vs. 0.23 ± 0.14 U/l
							–	11 ± 4 → 29 ± 7 mmHg/l/min		AEC apoptosis: 14 ± 3 vs. 6 ± 1 cells/mm^2^
Kuo and Liao (2001)	1–7	20	3	12 → 24/4		4	–	22 ± 2 → 33 ± 4		–
								1,210 ± 240 → 2,650 ± 450 dyne*s*cm^−5^		
Kuo (2001)	1–7	20	3	12 → 24/4	–	4	–	22 ± 3 → 33 ± 3	BQ-123 8 mg iv^∫^	Blood endothelin-1: no difference (?)
							–	1,246 ± 274 → 2,591 ± 545 dyne*s*cm^−5^		
Tollofsrud et al. (2001)	4–12	11	3	18/3 → 23 ± 3	10–15	2	1.2 ± 0.2 → 0.8 ± 0.1	17 ± 3 → 26 ± 3	F_i_O_2_: 0.21 vs. 1.0	Blood hypoxanthin: 56 ± 20 vs. 38 ± 10 μmol/l
							73 ± 2 → 104 ± 12	0.02 ± 0.02 → 0.04 ± 0.02		
Tølløfsrud et al. (2002)	0–2	11	3	20/3	10–15	8	1.4 ± 0.2 → 0.9 ± 0.3	23 ± 4 → 33 ± 6	F_i_O_2_, albumin	Endothelin-1: 2.4 ± 1.0 vs. 2.1 ± 0.7 ng/l
							88 ± 5 → 110 ± 40	0.10 ± 0.05 → 0.18 ± 0.08		
Dargaville et al. (2003)	14	20	4	15–20/4	–	5	0.9 ± 0.1 → 0.4 ± 0.1	–	Surfactant and perfluorocarbon	Protein: 7.1 ± 3.2 vs. 4.7 ± 1.9 vs. 8.2 ± 3.8 mg/ml
							75 ± 3 → 122 ± 8	–		Albumin: 3.3 ± 2.3 vs. 1.9 ± 1.1 vs. 4.1 ± 2.9 mg/ml
										DPPC: 0.6 ± 0.5 vs. 1.0 ± 0.3 vs. 1.1 ± 0.5 mg/ml
Hilgendorff et al. (2003)	1–11	20	5	15 → 25/2 → 4	?	5.5	–	–	rSP-C surfactant	Tissue IL-1β: 1.0 ± 0.3 vs. 0.2 ± 0.3 aU^‡^
										Tissue IL-6: 1.0 ± 0.3 vs. 2.1 ± 0.4 aU
										Tissue IL-8: 1.0 ± 0.2 vs. 0.4 ± 0.2 aU
										Tissue TGF-β: 1.0 ± 0.4 vs. 1.0 ± 0.2 aU
										Tissue IL-10: 1.0 ± 0.6 vs. 3.5 ± 0.5 aU
Korhonen et al. (2003)	1–3	6.5	1.5	20/4	–	12	–	–	Surfactant	MPO: 0.5 ± 0.2 vs. 0.8 ± 0.2 U/mg protein
										Protein: 1.4 ± 0.7 vs. 2.6 ± 0.4 mg/ml
										TNF-α: 121 ± 20 vs. 157 ± 33 pg/ml
										PLA_2_: 8 ± 6 vs. 401 ± 91 U/l
Castellheim et al. (2004)	0–2	13.5	4	–	–	5	–	–	None	Blood C5b-9: 0.3 vs. 1.5–2.4 U/ml
Korhonen et al. (2004)	0–2	6.5	1.5	20/4	–	12	–	–	Pentoxifylline 20 mg/kg iv	TNF-α: 42 ± 22 vs. 17 ± 5 pg/ml
										Protein 1.1 ± 0.3 vs. 0.6 ± 0.2 mg/ml
										Tissue MPO: 1.6 ± 1.0 vs. 1.5 ± 0.2
Lindenskov et al. (2004)	0–2	13.5	5	/5	13–15	5	2.2 ± 0.1 → 1.2 ± 0.1	–	None	Blood C5b-9: +82 ± 34%
										Blood IL-1β: 25 ± 48 → 112 ± 50 pg/ml
										Blood TNF-α: 55 ± 28 → 128 ± 15% increase
Shekerdemian et al. (2004)	?	20	3	?	–	6	–	18 ± 1 → 24 ± 1	BQ-123 1 mg/kg iv^∫^	Blood endothelin-1: 2.2 ± 0.4 vs. 2.9 ± 0.3 pg/ml
							–	65 ± 5 → 106 ± 10 mU/kg		
Tølløfsrud et al. (2004)	0–2	11	3	–	–	8	–	–	Albumin it	IL-8: 17 ± 13 vs. 94 ± 57 pg/ml
van Kaam et al. (2004a)	35 ± 15 h	14	10	8–10/2 → 15–22/4	–	6	–	–	CV vs. HFO^π^	MPO: 0.7 ± 0.1 vs. 0.5 ± 0.1 U/ml
Castellheim et al. (2005)	0–2	13.5	4	<45/?	12 ± 4	7	2.2 ± 0.4 → 1.0 ± 0.3	–	None	Blood C5b-9: 0.2 ± 0.1 vs. 3.8 ± 1.8 aU
							–	–		Blood IL-6: 40 ± 60 vs. 460 ± 390 pg/ml
										Blood IL-8: 20 ± 4 vs. 26 ± 6 pg/ml
										Blood CD11/18: not different (?)
Holopainen et al. (2005)	10–12	6.5	3	/2–3	16–20	6	–	–	ivIg 0.8 g/kg^Δ^	MPO: 11 ± 3 vs. 215 ± 58 mU/g protein
							–	(+165%)		PLA_2_: 0.15 ± 0.07 vs. 0.10 ± 0.03 U/g
Lindenskov et al. (2005)	0–2	13.5	4	<45/?	–	8	−46–60%	21 ± 2 → 32 ± 10	Albumin	IL-8: 9.5 ± 1.6 vs. 9.6 ± 0.4 ng/ml
							–	–		Protein 3.5 ± 0.3 vs. 3.6 ± 0.5 mg/ml
Hilgendorff et al. (2006)	1–11	20	5	15 → 25/2 → 4	–	5.5	2.2 ± 0.2 → 0.7 ± 0.1	–	rSP-C surfactant	Tissue SP-B: 0.1 (0.1–0.4) vs. 0.6 (0.1–1.0) 2^∧^ΔΔct
										Tissue SP-C: 0.5 (0.3–0.5) vs. 0.5 (0.1–0.8) 2^∧^ΔΔct
Jeng et al. (2006)	<14	25	3–5	10–13/3	10	4	1.1 ± 0.2 → 0.4 ± 0.1	–	Surfactant and liquid ventilation	Blood IL-1β: 2.3 ± 0.3 vs. 0.1 ± 0.02 ng/ml
										Blood IL-6: 1.6 ± 0.4 vs. 0.1 ± 0.03 ng/ml
Angert et al. (2007)	1–3	20	3	/3	–	24	–	–	rhCC10 5 mg/kg it^¢^	IL-8: 4.8 ± 1.9 vs. 5.5 ± 2.8 ng/mg protein/ml
										TNF-α: 1.3 ± 0.6 vs. 0.5 ± 0.3 ng/mg protein/ml
Salvesen et al. (2008)	0–2	13.5	4	18–20/4	8–14	6	2.1 ± 0.2 → 0.9 ± 0.3	–	Albumin 0.6 g/kg it	Blood sC5b-9: 0.2 ± 0.7 vs. 0.9 ± 1.6 aU/ml
							59 ± 5 → 69 ± 24	–		Blood TNF-α: 82 ± 19 vs. 62 ± 7 pg/ml
										Blood IL-1β: 29 ± 75 vs. 83 ± 44 pg/ml
										Blood IL-6: 32 ± 95 vs. 50 ± 87 pg/ml
Saugstad et al. (2008)	2–5	?	3–4	?	10–15	8	–	–	Albumin it	IL-8: 93 vs. 18 pg/ml
Wang et al. (2010)	7–14	20	3–5	17 → 27/5	10	4	1.4 ± 0.2 → 0.6 ± 0.1	–	Surfactant	IL-1β: 265 ± 61 vs. 65 ± 18 ng/ml
										IL-6: 0.6 ± 0.4 vs. 0.1 ± 0.1 μg/ml
										TNF-α: 0.4 ± 0.1 vs. 0.4 ± 0.1 μg/ml
										AEC apoptosis: 22 ± 6 vs. 8 ± 4 per power field
Salvesen et al. (2014)	0–2	10	4.5	18–20/5	6–12	6	2.0 ± 0.2 → 0.8 ± 0.2	–	Poractant alpha and CHF5633^¬^	Blood lipid peroxidation: 1.5 ± 0.4 vs. 0.4 ± 0 nmol/mg
										Blood sC5b-9: 0.8 ± 0.4 vs. 1.1 ± 0.5 aU/ml
										Blood TAT~: 50 ± 29 vs. 145 ± 81 μg/ml
										Blood PAI-1^¤^: 145 ± 42 vs. 72 ± 25 ng/ml
										Blood TNF-α: 90 ± 21 vs. 150 ± 25 pg/ml
										Blood IL-6: 0.2 ± 0.06 vs. 0.6 ± 0.3 ng/ml
										Blood IL-1β: 0.18 ± 0.04 vs. 0.11 ± 0.04 ng/ml
Lin et al. (2016)	<14	25	6–7	11 → 20/5	8	6	1.3 ± 0.1 → 0.6 ± 0.1	–	Surfactant and budenoside	IL-1β: 2.1 (2.0–3.1) vs. 0.8 (0.3–1.3) ng/ml
							–	–		IL-6: 2.7 (2.4–2.8) vs. 1.3 (2.1–0.7) ng/ml
										IL-8: 2.8 (2.1–4.6) vs. 1.7 (0.4–3.3) ng/ml
Lin et al. (2017)	4–12	25	6	15/5 → 23 ± 1	–	8	0.9 ± 0.1 → 0.6 ± 0.01	–	Dexa, budenoside iv	Tissue lung injury score:↓
Thomas et al. (2018)	2	9.9	5.5	/5	–	5	–	–	Anti-CD14, anti-C5a	Blood IL-1β: 240 ± 45 vs. 172 ± 52 pg/ml
										Blood IL-6: 154 ± 38 vs. 181 ± 34 pg/ml
										MPO: 856 ± 35 vs. 265 ± 30 ng/ml

* PIP/PEEP, peak inspiratory pressure/positive end-exspiratory pressure;

#V_T_, tidal volume;

$ study l., study length;

§C_rs_/R_rs_, compliance/resistance of the respiratory system;

¶ PAP/PVR(I), pulmonary arterial pressure/pulmonary vascular resistance (index);

† a.o., and others;

∫ BQ-123, endothelin antagonist;

‡ aU, arbitrary units;

π CV/HFO, conventional ventilation/high-frequency oscillation;

Δ ivIg, intravenous immunoglobulin;

¢ rhCC10 it, recombinant human Clara Cell protein 10, intratracheally administered;

¬ *CHF5633, synthetic surfactant containing SP-B/C, DPPC, POPG; ^~^TAT, thrombin antithrombin complex; ^¤^PAI-1, plasminogen activator inhibitor-1*.

*PIP/PEEP, C_rs_/R_rs_, PAP/PVR(I), immunologic response: arrow (→) delineates changes by the lung injury protocol (i.e., meconium instillation into the airways); vs. delineates differences secondary to a specific intervention (first place control group, second place intervention group data)*.

*Immunologic response parameters are from broncho-alveolar lavage fluid (BALF) unless specified otherwise*.

ROS are inflammatory mediators protecting the host from external damage, however, they simultaneously inherit a strong potential to harm the host in case of overwhelming activation. The complement system is linked with C5a-mediated leukocyte oxidative burst (Castellheim et al., 2005) and plays an important role by the supply of C5b-9 which has the potential to also directly attack alveolar epithelial cells. While the combined application of C5a- and CD14-inhibitors resulted in a pronounced attenuation of inflammatory parameters (particularly IL-1β and MPO), the clinical course of the intervention group was not different from the control group (Thomas et al., 2018).

Meconium has high concentrations of phospholipase A_2_ (sPLA_2_), a family of ubiquitous enzymes that release arachidonic acid by the cleavage of membrane phospholipids or surfactant (Holopainen et al., 1999a; De Luca et al., 2009). The administration of dexamethasone which reduces a stimulated sPLA_2_ synthesis (Hoeck et al., 1993), does not contain sPLA_2_ activity nor reduce inflammation in the newborn piglet model (Holopainen et al., 2001).

By far the majority of the studies (Table 11) focus on the effect of surfactant substitution for improvements in lung mechanics and gas exchange. While surfactant is known to protect the lungs from inflammation modulating peroxidation, formation of nitric oxide, sPLA_2_, eicosanoids, and cytokines (Wright, 2003), some surfactant fractions, such as palmitoyl-oleoyl-phosphatidylglycerol (POPG) (Numata et al., 2010; Spengler et al., 2018) and dioleoyl-phosphatidylglycerol (DOPG) (Preuß et al., 2014) exert potent anti-inflammatory action and deserve further research (Salvesen et al., 2014). The administration of a POPG-based synthetic surfactant (CHF5633), however, did not improve the clinical outcome in the newborn piglet model not reflecting some marked inflammatory mediator attenuations, such as reductions in IL-1β and lipid peroxidation (Salvesen et al., 2014).

### Lavage Model

The lavage model (Table 12) excels by the fine-tuning of impairment of gas exchange (oxygenation index, ventilation efficiency index), lung mechanics (compliance and resistance of the respiratory system), and lung volumes (alveolar volume, functional residual capacity). Once an appropriate lung injury has been set (mostly monitored by reductions of oxygenation and compliance) the piglet remains stable with regard to circulation and other organ system function. By the use of continuous sedation/analgesia, mechanical ventilation can be perpetuated for several days allowing further injury to the lungs (double-/triple hit injury models) or specific interventions. That way, the requirements for NARDS by the Montreux definition (acute onset; diffuse, bilateral, irregular opacities; edema; oxygenation deficit) can be completely satisfied (De Luca et al., 2017). In addition, as an animal model of acute lung injury, physiologic changes (decreased compliance, reduced functional residual capacity, V/Q-abnormalities, impaired alveolar fluid clearance), biological changes (increased endothelial and epithelial permeability, increased cytokine concentrations in BALF or lung tissue, protease activation, coagulation abnormalities), and pathological changes (infiltration by PMNL, fibrin deposition and augmented intra-alveolar coagulation, denudation of the basement membrane) can be observed (Matute-Bello et al., 2008).

**Table 12:** Lavage model.

**References**	**Age (days)**	**Lavages (n)**	**Volume (ml/kg)**	PIP/PEEP^*^ (mbar)	**${\text{V}}_{\text{T}}^{\#}$ (ml/kg)**	**study l.^**$**^ (h)**	**${\text{C}}_{\text{rs}}^{§}$ (ml/mbar/kg)**	${\text{R}}_{\text{rs}}^{¶}$ (mbar/l*sec)	**FRC^†^ (ml/kg)**	**Intervention**	**Immunologic response**
Sood et al. (1996a)	4–9	?	35	17 → 29/6	–	1.5	1.3 ± 0.1 → 0.5 ± 0.1	32 ± 3 → 58 ± 3	29 ± 1 → 12 ± 2	Beractant, DPPC, and KL4^‡^	Protein: 347 ± 142 vs. 56 ± 13 mg/dl (KL4) histopathology scores: no difference
Abubakar et al. (1998) (double-hit: lavage and overventilation)	<3	?	35	15 → 40 → ?/3 → 2 → 4	10–13	24	–	–	–	Heparin, ATIII	ATIII: 49 ± 8 vs. 57 ± 10 μg/ml
											^125^I-fibrinogen uptake: 19 ± 10 vs. 10 ± 9%
											lung injury score: 1.99 ± 0.71 vs. 1.55 ± 0.63
Balaraman et al. (1998)	4–8	13 ± 1 + 4	35	? → 32/6	–	4	? → 0.7 ± 0.1	?	?	(un)diluted DPPC	Protein: 25 ± 6 vs. 15 ± 9 mg/dl (undil. vs. dil.)
Jeng et al. (2002)	1–14	?	30	/5	15	3	1.7 ± 0.2 → 0.8 ± 0.1	–	–	FC-77^π^	Alveolar inflammation score: 1.8 ± 04 vs. 0.6 ± 0.3
											AEC^Δ^ necrosis score: 3.1 ± 0.5 vs. 1.4 ± 0.3
Merz et al. (2002)	1–3	?	30	16 → 20/2 → 4	?	24	–	–	–	Surfactant, HFOV and liquid ventilation	LTB_4_: 1.5 ± 0.3 vs. 1.1 ± 0.2 ng/ml
											IL-6: 1.2 ± 0.3 vs. 1.6 ± 0.9 ng/ml
											TNF-α: 1.3 ± 0.4 vs. 1.0 ± 0.3 ng/ml
von der Hardt et al. (2002)	?(4 kg)	?	30	20 → 32/4 → 8	–	6	–	–	–	FC-77	Tissue IL-1β: 15 ± 4 vs. 1.4 ± 0.4 rU¢
											Tissue IL-6: 1.0 ± 0.2 vs. 0.4 ± 0.2 rU
											Tissue IL-8: 2.4 ± 0.6 vs. 0.7 ± 0.3 rU
											Tissue TGF-β: 1.7 ± 0.2 vs. 1.2 ± 0.1 rU
van Kaam et al. (2003b)	0–2	?	50	10–12 → 25/2 → 5 → 10	–	5	–	–	–	OLC^¬^-ventilation	Cells: 0.8 ± 0.5 vs. 0.4 ± 0.2 × 10^6^/ml
											IL-8: not different
											TNF-α: not different
											Thrombin activity: not different
van Kaam et al. (2003a)	0–2	?	50	9–12 → 25/2 → 5 → 15	–	5	–	–	–	OLC-ventilation	Protein: 0.7 ± 0.2 vs. 1.0 ± 0.1
van Kaam et al. (2004a)	?	?	50	25/4–5 → 10	7	5	–	–	TLC^∫^: 57 ± 20 → 22 ± 6	OLC-ventilation on GBS it^¬^	Bacterial infiltration score: 11 ± 1 vs. 4 ± 1
											Cellular infiltration score: 11 ± 1 vs. 6 ± 1
van Kaam et al. (2004b)	0–2	15 ± 5	50	8–10/2 → 10	–	5	–	–	–	HL10 surfactant and OLC-ventilation	Protein: 0.8 ± 0.1 vs. 0.3 ± 0.1 mg/ml
											SA/LA-ratio~: 1.6 ± 0.4 vs. 0.2 ± 0.1
											IL-8: 15 ± 7 vs. 41 ± 20 pg/ml
											Cells: 8 ± 5 vs. 1 ± 1 × 10^6^/ml
van Kaam et al. (2005)	?	?	50	/5	7	5	–	–	–	HL10 surfactant and OLC-ventilation on GBS it	IL-8: 19 (5–44) vs. 4 (0–6) ng/ml
											MPO: 8 (3–22) vs. 4 (0–7) ng/ml
											TNF-α: 1.2 (0–1.7) vs. 1.4 (0–3.2) ng/ml
Krause et al. (2005)	2–10	12 ± 5	30	23/4 → 8	6	6	0.9 ± 0.4 → 0.5 ± 0.2	47 ± 7 → 77 ± 13	24 ± 3 → 9 ± 2	Poractant	IL-6: 30 ± 29 vs. 16 ± 10 U/ml
											IL-8: 0.4 ± 0.2 vs. 1.0 ± 0.6 ng/ml
											TNF-α: 0.64 ± 0.69 vs. 1.42 ± 1.37 U/ml
											Protein: 53 ± 7 vs. 50 ± 18 mg/l
Ankermann et al. (2005b)	2–10	9–12	30	→23–27/4	6	6	1.9 ± 0.3 → 0.6 ± 0.2	52 ± 7 → 74 ± 14	24 ± 4 → 10 ± 3	Poractant and anti-IL-8 AB	IL-8: 0.3(0.1–0.7) vs. 0.8(0.4–2.3) vs. 3.4(0.6–16.1) ng/ml
											IL-6: 29 ± 28 vs. 16 ± 10 vs. 199 ± 458 U/ml
											TNF-α: 0.6 ± 0.6 vs. 1.4 ± 1.3 vs. 3.7 ± 4.9 U/ml
Ankermann et al. (2005a)	2–10	10 ± 4	30	→23–5/4	6	6	1.8 ± 0.3 → 0.5 ± 0.2	50 ± 8 → 91 ± 17	27 ± 6 → 12 ± 2	Poractant and IKK-NBD peptide^¤^	Protein: 50 ± 5 vs. 38 ± 5 mg/l
											IL-1β: 0.09 ± 0.08 vs. 0.06 ± 0.05 U/ml
											IL-8: 2.3 ± 1.1 vs. 2.2 ± 1.0 ng/ml
											TNF-α: 2.9 ± 3.0 vs. 1.3 ± 0.9 U/ml
											LTB_4_: 3.5 ± 1.4 vs. 2.0 ± 0.6 pg/ml
Ankermann et al. (2006)	2–10	11 ± 3	30	→25/4 → 8	6	6	1.7 ± 0.4 → 0.5 ± 0.2	–	25 ± 4 → 10 ± 4	Poractant and MK886^▵^	LTB_4_: 3.5 ± 1.4 vs. 2.3 ± 1.6 pg/ml
											IL-8: 1.0 ± 0.6 vs. 4.7 ± 5.4 μg/ml
											Cells: 625 ± 36 vs. 525 ± 176/μl
van Veenendaal et al. (2006)	<7	12 ± 4	50	10 → 26/2 → 6 or 10	7–8	4	–	–	–	HL-10 surfactant and open lung ventilation	Protein: 1.6 ± 0.4 vs. 0.5 ± 0.2 mg/ml
											IL-8: 1.8 (0–44) vs. 0 (0–0) ng/ml
											MPO: 0.6 (0–2.1) vs. 0 (0–0) ng/ml
von Bismarck et al. (2007)	2–5	20 ± 6	30	→24/6	7	24	0.8 ± 0.2 → 0.3 ± 0.1	–	30 ± 7 → 15 ± 4	HL-10 surfactant and IKK-NBD peptide	Protein: 747 (621–1268) vs. 1,020 (145–1798) vs. 1,322 (909–2,790) mg/l
											Tissue MPO: 0.45 ± 0.16 vs. 0.38 ± 0.22 vs. 0.26 ± 0.21 U/mg
											LTB_4_: 78 ± 74 vs. 65 ± 54 vs. 23 ± 17 pg/ml
											Tissue NF-κB: 1.0 ± 0.1 vs. 0.9 ± 0.2 vs. 0.7 ± 0.1 aU^∧^
											Tissue aSMase^⌞^–activity: 25 ± 1 vs. 22 ± 2 vs. 16 ± 2 nmol/mg/h
											Tissue ceramide: 544 ± 40 vs. 455 ± 59 vs. 358 ± 64 pmol/g
von Bismarck et al. (2008)	2–5	21 ± 8	30	→25/6	7	24	1.0 ± 0.4 → 0.25 ± 0.01	–	34 ± 4 → 23 ± 12	HL-10 surfactant and imipramine 5 mg it	Tissue aSMase-activity:27 ± 2 vs. 20 ± 2 vs. 17 ± 1 nmol/mg/h
											Tissue ceramide: 578 ± 27 vs. 464 ± 43 vs. 494 ± 38 pmol/g
											Tissue NF-κB: 1.0 ± 0.4 vs. 0.5 ± 0.1 vs. 0.7 ± 0.2 aU
											LTB_4_: 95 ± 72 vs. 54 ± 22 vs. 32 ± 13 pg/ml
											IL-8: 455 ± 320 vs. 125 ± 134 vs. 56 ± 40 pg/ml
Chada et al. (2008)	9–12	–	30	20 → 26/4 → 6	7	12	1.4 ± 0.1 → 0.3 ± 0.05	–	–	Anakinra 100 mg aerosol vs. iv	Lung injury score: 14 ± 3 vs. 12 ± 4 vs. 10 ± 4
											Tissue IL-1β: 4.9 ± 2.4 vs. 0.9 ± 0.3 vs. 1.9 ± 0.6 rU
											Tissue IL-8: 5.0 ± 1.6 vs. 1.3 ± 0.4 vs. 2.2 ± 0.5 rU
von Bismarck et al. (2009)	2–5	21 ± 2	30	→25/6	7	24	0.90 ± 0.15 → 0.33 ± 0.04	–	33 ± 8 → 16 ± 2	HL-10 surfactant, dexamethasone, IKK-NBD peptide	IL-8: 2,093 ± 583 vs. 491 ± 144 vs. 351 ± 117 pg/ml
											LTB_4_: 78 ± 31 vs. 71 ± 11 vs. 23 ± 7 pg/ml
											NF-κB activity: 100 ± 4 vs. 55 ± 3 vs. 32 ± 5%
Yang et al. (2010)	<14	–	10	/5	10	4	1.07 ± 0.17 → 0.51 ± 0.05	–	–	Beractant and budenoside	AEC necrosis score: 4 (4) vs. 2.5 (2–3) vs. 2.5 (2–3)
											Lung injury score: 27.5 vs. 15 vs. 14
Preuß et al. (2012b) (triple-hit: lavage overventilation LPS)	3–6	15 ± 2	30	/6	7	72	1.3 ± 0.2 → 0.5 ± 0.2	51 ± 11 → 99 ± 40	–	Poractant alfa and IP3^♢^	Tissue aSMase activity: 2.6 ± 0.3 vs. 1.9 ± 0.2 vs. 1.6 ± 0.2 nmol/g/h
											Tissue ceramide: 2.4 ± 0.4 vs. 2.1 ± 0.3 vs. 1.6 ± 0.4 nmol/mg
											Tissue caspase-8: 100 vs. 76 ± 8 vs. 60 ± 14%
											AEC apoptosis: 15 ± 2 vs. 14 ± 2 vs. 7 ± 2%
											Tissue amphiregulin: 1.0 ± 1.6 vs. 1.5 ± 2.1 vs. 0.1 ± 0.1 rU
											Tissue TGF-β1: 1.0 ± 0.8 vs. 0.9 ± 0.6 vs. 0.02 ± 0.01 rU
											Tissue IL-6: 1.0 ± 0.5 vs. 2.0 ± 1.3 vs. 0.05 ± 0.01 rU
											CD14^+^/18^+^: 281 ± 61 vs. 264 ± 38 vs. 116 ± 16 × 10^3^/μl
Preuß et al. (2012a) (triple-hit: lavage overventilation LPS)	3–6	16 ± 3	30	/6	7	72	1.2 ± 0.2 → 0.5 ± 0.2	57 ± 18 → 109 ± 49	–	Poractant alfa and PIP2°	Cells: 555 ± 238 vs. 379 ± 179 vs. 149 ± 130 × 10^3^/μl
											CD14^+^/18^+^: 331 ± 96 vs. 244 ± 46 vs. 99 ± 30 × 10^3^/μl
											Tissue amphiregulin: 1.0 ± 1.3 vs. 1.8 ± 2.2 vs. 0.1 ± 0.1 rU
											Tissue TGF-1β: 1.0 ± 0.6 vs. 0.9 ± 0.7 vs. 0.02 ± 0.01 rU
											Tissue IL-6: 1.0 ± 0.5 vs. 2.3 ± 1.4 vs. 0.003 ± 0.001 rU
											Tissue aSMase activity: 2.6 ± 0.3 vs. 1.9 ± 0.2 vs. 1.8 ± 0.2 nmol/g protein/h
											Tissue ceramide: 2.4 ± 0.4 vs. 2.1 ± 0.2 vs. 2.0 ± 0.5 nmol/mg
Qin et al. (2013)	?	?	35	10 → 20/2 → 4	?	48	–	–	–	CMV vs. HFOV^⋂^	Lesser AEC migration, lesser giant lamellar bodies, lesser vacuoles and cell polymorphisms
Fu et al. (2013)	0–3	–	35	10 → 20/2 → 4	?	48	–	–	–	CMV vs. HFOV	Alveolar macophages: 76 ± 14 vs. 69 ± 8%
											Alveolar red blood cells: 380 ± 15 vs. 230 ± 18/100 alveoli
Yang et al. (2013)	7–14	?	10	/5	8	24	0.74 ± 0.03 → 0.46 ± 0.02	–	–	Surfactant and Budenoside	IL-1β: 27 ± 7 vs. 30 ± 13 pg/ml
											TNF-α: 1.0 ± 0.2 vs. 0.9 ± 0.2 ng/ml
											Lung injury score: 15 (12–18) vs. 12 (7–13)
Preuß et al. (2014) (triple-hit: lavage overventilation LPS)	3–6	15 ± 1	30	/6	7	72	1.16 ± 0.31 → 0.30 ± 0.06	60 ± 18 → 112 ± 52	–	Poractant alfa and DOPG^ʁ^	sPLA_2_: 2.0 ± 0.3 vs. 0.9 ± 0.2 vs. 0.2 ± 0.2 pg/ml
											TNF-α: 0.24 ± 0.05 vs. 0.19 ± 0.01 vs. 0.10 ± 0.01 pg/ml
											Cells: 448 ± 222 vs. 457 ± 228 vs. 73 ± 79 × 10^3^/μl
											CD14^+^/18^+^: 287 ± 58 vs. 340 ± 65 vs. 52 ± 6 × 10^3^/μl
											Tissue amphiregulin: 1.0 ± 1.7 vs. 0.6 ± 0.7 vs. 0.1 ± 0.2 rU
											Tissue TGF-β1: 1.0 ± 0.8 vs. 1.4 ± 0.8 vs. 0.02 ± 0.01 rU
											AEC apoptosis: 30 ± 13 vs. 35 ± 10 vs. 15 ± 6 of 200 AEC
Spengler et al. (2018) (triple-hit: lavage overventilation LPS)	2–7	17 ± 2	30	/6	7	72	1.2 ± 0.4 → 0.4 ± 0.1	71 ± 19 → 150 ± 62	–	Poractant alfa and IP3, PIP2, POPG^□^, ^ʁ^DOPG	Tissue aSMase activity: 49 ± 4 vs. 42 ± 5 vs. 20 ± 3 vs. 32 ± 2 vs. 40 ± 7 vs. 40 ± 3 nmol/mg/h
											Tissue ceramide: 1.2 ± 0.2 vs. 1.1 ± 0.1 vs. 0.6 ± 0.1 vs. 1.0 ± 0.1 vs. 1.1 ± 0.1 vs. 1.1 ± 0.1 pmol/mg
											Tissue NLRP3^∞^: 100 ± 7 vs. 91 ± 9 vs. 32 ± 9 vs. 87 ± 6 vs. 82 ± 12 vs. 81 ± 9%
											Tissue caspase-1: 100 ± 5 vs. 91 ± 23 vs. 35 ± 13 vs. 88 ± 16 vs. 103 ± 21 vs. 84 ± 18%

*Further data from (Spengler et al., 2018): tissue IL-1β: 100 ± 11 vs. 99 ± 17 vs. 33 ± 16 vs. 86 ± 24 vs. 92 ± 19 vs. 75 ± 16%, Tissue IL-18: 100 ± 12 vs. 99 ± 17 vs. 34 ± 8 vs. 97 ± 17 vs. 95 ± 14 vs. 80 ± 24%, Tissue IκBα: 8 ± 4 vs. 12 ± 7 vs. 66 ± 16 vs. 50 ± 15 vs. 31 ± 9 vs. 26 ± 6%, Tissue IκB-kinase: 99 ± 4 vs. 88 ± 4 vs. 23 ± 12 vs. 33 ± 11 vs. 54 ± 21 vs. 65 ± 15%, Tissue TGF-β1: 103 ± 2 vs. 97 ± 7 vs. 36 ± 12 vs. 95 ± 24 vs. 93 ± 25 vs. 85 ± 34%, Tissue IFN-γ: 100 ± 13 vs. 90 ± 13 vs. 26 ± 15 vs. 89 ± 21 vs. 94 ± 14 vs. 76 ± 21%, Tissue elastin: 101 ± 5 vs. 97 ± 6 vs. 49 ± 5 vs. 85 ± 7 vs. 83 ± 14 vs. 51 ± 8%, Tissue MMP-1^≡^: 101 ± 6 vs. 91 ± 13 vs. 43 ± 7 vs. 65 ± 17 vs. 80 ± 17 vs. 52 ± 12%, Albumin: 378 ± 36 vs. 368 ± 16 vs. 109 ± 15 vs. 357 ± 24 vs. 327 ± 16 vs. 124 ± 9 μg/ml, Blood SP-D: 326 ± 19 vs. 326 ± 13 vs. 65 ± 7 vs. 288 ± 16 vs. 260 ± 17 vs. 126 ± 6 ng/ml, AEC apoptosis: 15 ± 5 vs. 21 ± 6 vs. 12 ± 2 vs. 11 ± 3 vs. n.d. vs. 9 ± 3%, MMP-8^≡^: 26 ± 8 vs. 24 ± 4 vs. 4 ± 0.5 vs. 23 ± 7 vs. 25 ± 8 vs. 5 ± 1 ng/ml, sPLA_2_: 20 ± 10 vs. 15 ± 6 vs. 13 ± 11 vs. 12 ± 11 vs. 28 ± 8 vs. 27 ± 9 pg/ml*.

* PIP/PEEP, peak inspiratory pressure/positive end-exspiratory pressure; ^#^V_T_, tidal volume;

$ study l., study length; ^§^C_rs_, compliance of the respiratory system: ^¶^Rrs, resistance of the respiratory system;

† FRC, functional residual capacity;

‡ KL4, synthetic surfactant containing DPPC and a synthetic SP-B derivate;

π FC-77, perfluorocarbon for liquid ventilation;

Δ AEC, alveolar epithelial cells; ¢rU, relative units;

∫ TLC, total lung capacity;

¬ OLC-ventilation on GBS it, open lung concept ventilation secondary to additional intratracheal instillation of group B streptococci; ~ SA/LA-ratio, small aggregate/large aggregate surfactant ratio;

¤ IKK-NBD peptide, NF-κB antibody; ^△^MK886, 5-lipoxygenase inhibitor;

∧ aU, arbitrary units; ^⌞^aSMase, acid sphingomyelinase;

♢ IP3, myo-inositol-1,2,6-trisphosphate;

° PIP2, L-α-phosphatidyl-L-myo-inositol-3,5-bisphosphate;

⋂ CMV/HFOV, conventional mechanical ventilation/high-frequency oscillatory ventilation;

ʁ DOPG, 18:1/18:1-dioeoyl-phosphatidylglycerol;

□ POPG, palmitoyl-oleoyl-phosphatidylglycerol;

∞ *NLRP3, nucleotide-binding domain, leucine-rich repeat-containing protein-3 (inflammasome); ^≡^MMP-1/8, matrix metalloproteinase-1; n.d., not determined*.

*PIP/PEEP, C_rs_, R_rs_, FRC, immunologic response: arrow (→) delineates changes by the lung injury protocol (i.e., repeated airway lavage); vs. delineates differences secondary to a specific intervention (first place control group, second place intervention group data)*.

*Immunologic response parameters are from broncho-alveolar lavage fluid (BALF) unless specified otherwise*.

The washout of endogenous surfactant by the use of warmed normal saline with a volume exceeding FRC (i.e., >25 ml/kg) ensures—in contrast to other models—acute onset of NARDS which is sustained by secondary damage to the remaining surfactant within the lung and the triggering of a marked inflammatory response which primarily activates all mechanisms of the innate immunity. Thus, the saline for lavage may be considered as a pathogen-associated molecular pattern (PAMP) being recognized by the collectin, ficolin, and pentraxin families which can act as opsonins either directly or by activating the complement system (as also shown in the meconium aspiration model) (Male, 2006). The innate immunity's response brings leukocytes and plasma proteins to the site of (lung) tissue damage. The arrival of leukocytes in the lung depends on chemokines and adhesion molecules expressed by the pulmonary vasculature endothelium, by the alveolar epithelium, and by the activity of local macrophages. Notably TNF-α, which is primarily produced by macrophages, induces the expression of adhesion molecules and chemokines, and may elicit the activation of NF-κB and apoptosis. Next to TNF-α, IL-1β plays an important part in the induction of adhesion molecules on the endothelium.

In this context it is surprising that many aspects of dampening the innate immune system in the overwhelming response of the neonatal lung secondary to repeated airway lavage have not been studied yet (Table 12). While the early studies considering immunologic aspects measured protein content in BALF, histopathology scores, and the concentrations of TNF-α, LTB_4_, IL-1β, and MPO as a surrogate parameter for neutrophil infiltration (Sood et al., 1996a; Merz et al., 2002; van Kaam et al., 2005), more recent studies put light on the selective inhibition of NF-κB (von Bismarck et al., 2007), IL-1β metabolism (Chada et al., 2008), general immune suppression by dexamethasone or budenoside (von Bismarck et al., 2009; Yang et al., 2010), eicosanoid suppression (Ankermann et al., 2006), and blockade of IL-8 (Ankermann et al., 2005b). The impact of the ceramide metabolism in nARDS has been investigated (Barnes, 2004; von Bismarck et al., 2008; Spengler et al., 2018), and the important role of NLRP3 (nucleotide-binding domain, leucine-rich repeat-containing protein-3) in a triple-hit lung injury model has been studied (Dos Santos et al., 2012; Spengler et al., 2018).

### Miscellaneous Models

NARDS-like severe pneumonia has been established by few authors, however, the maintenance of HR and BP as prerequisite for a stable model of primary lung injury—in contrast to e.g., the rabbit model—can also be demonstrated in direct GBS instillation into the airways (van Kaam et al., 2004b). The selective inoculation of GBS into the lower lobes of newborn piglets resulted in widespread alveolar atelectasis, loss of hyaluronan, and an increased systemic uptake of the microorganisms into the circulation (Juul et al., 1996). Using isolated selectively perfused piglet lungs, an increase in total pulmonary resistance is observed following GBS-instillation into the pulmonary circulation (Aziz et al., 1993). Intravenous E. coli endotoxin has been used to induce a moderate impairment in oxygenation and lung mechanics without experiencing any positive changes by surfactant application (Sood et al., 1996b).

Moderately acute lung injury models involving mechanical ventilation have been established testing the effects of overventilation with and without the application of 100% O2 (Davis et al., 1989, 1991; Ehlert et al., 2006). Dexamethasone (Davis et al., 1995), G-CSF (Wolkoff et al., 2002), recombinant human Clara Cell protein 10 (rhCC10) intratracheally for hyperoxia (Chandra et al., 2003), and nitric oxide (NO) (Youssef et al., 1999) have been assessed using increased observation times for several days prior to mechanical ventilation.

An exceptional model of wood smoke inhalation treated with surfactant and partial liquid ventilation has been set up by Jeng et al. (2003).

### Perspective: Newborn Animal Models of NARDS Involving Mechanical Ventilation

Many more newborn animal models from different species and subject to a variety of acute lung injury protocols have been set up (Table 13). While the clinical relevance varies considerably among species with regard to body size (piglets and lambs 70–80% of human size, rodents 0.1–1.6%), availability, and similarities with the human innate immunity, models with rodents have been used abundantly for low costs and genetic similarity among animals despite of their limitations in comparability with human newborns. Ethical considerations and very high costs limit the availability of newborn baboons which have been studied almost exclusively in preterm models of infant respiratory distress syndrome (IRDS). Thus, piglet and lamb models clearly head the list of established newborn animal models of NARDS also considering the wide variety of direct and of only one direct-to-indirect lung injury protocol (i.e., intraamniotic LPS administration, Table 13). With a body size being 70–80% of human newborns, a gravidity length of 40%, a thoracic-to-abdominal relationship of 1:1 as in humans (compared to a relationship of 1:2–3 in rodents), and a lung:body weight ratio of 1.6 (porcine) vs. 1.8 (human), the piglet model excels also taking into account the similarities of the innate immunity, such as an 80% accordance of the hypervariable region of the Toll-like receptor 4, LPS response, and production of nitric oxide.

**Table 13:** Newborn animal models of acute respiratory distress syndrome (NARDS) involving mechanical ventilation.

**Species**	**Availability**	**Costs**	**Clinical relevance**	**Max. period of mechanical ventilation (d)**	**Common types of lung injury (see below)**	TLR4 HVR^*^ (%)	**Pulmonary intravascular macrophages**	**LPS^**#**^ sensitivity**
Baboon	Scarce	Very high	Very high	28	IRDS^1^, HO^2^	95	Few^3^^$^	Intermediate
Pig	Good	Intermediate	High	4	L, OV, ITLPS, MAS, HO, IPI	~80^4^	Yes^5^	Intermediate^6^
Sheep	Seasonal	High	High	<1	IRDS, IT/ALPS, HCL^9^, MAS^10^, IPI^11^, HO^12^	83–85^7^	Yes^3^^,^^5^	High^8^
Rabbit	Good	Low	Low	<1	L, MAS^13^	57	No	Intermediate
Guinea pig	Good	Low	Low	<1	IRDS, HO	?	No	Low
Rat	Good	Low	Low	<1	HO^14^, OV^15^, HCL^15^, IPLPS^17^	48	No^$^	Low^16^

* TLR4 HVR, similarity with the human hypervariable region of Toll-like receptor 4 expressed in percent;

# LPS, lipopolysaccharide;

$ *few/no, pulmonary intravascular macrophages may be induced, such as in human kind*.

Common types of lung injury: IRDS, respiratory distress syndrome of the premature infant (surfactant deficiency, immature alveolarization, precursor of bronchopulmonary dysplasia); HO, hyperoxia/exposure to 100% O_2_ (epithelial injury, PMNL infiltration, epithelial-to-mesenchymal transition); L, lavage with normal saline (surfactant depletion, PMNL and macrophage infiltration, impaired lung volumes and lung mechanics); OV, overventilation (disruption of alveolar structures, epithelial-to-mesenchymal transition); ITLPS, intratracheal LPS (PMNL infiltration, cytokine storm), MAS, meconium aspiration syndrome (pulmonary hypertension, impaired lung mechanics, release of reactive oxygen species and sPLA_2_); IPI, infectious pathogen instillation (increased permeability, interstitial and alveolar edema, PMNL infiltration); IA/PLPS, intraamniotic/intraperitoneal LPS (PMNL infiltration, cytokine storm); HCL, hydrochloric acid lavage (disruption of alveolar and capillary structures, PMNL infiltration).

1 Yoder et al. (2000),

2 Lee et al. (2005),

3 Brain et al. (1999),

4 Palermo et al. (2009),

5 Cantu et al. (2006),

6 Spengler et al. (2018),

7 Hillman et al. (2008),

8 Polglase et al. (2008),

9 Cox et al. (2002),

10 Foust III et al. (1996),

11 Larios Mora et al. (2015),

12 Kumar et al. (2010),

13 Mokra et al. (2016),

14 Wu et al. (2019),

15 Sly et al. (2013),

16 Liu et al. (2013),

17 *Trummer-Menzi et al. (2012)*.

As we know that pathophysiologic peculiarities in ARDS of different age groups show considerable overlap in animal models (Schouten et al., 2015) as well as in human kind (Schouten et al., 2019), it is of uttermost importance to describe and to tackle the numerous facets of innate immunity in the various animal models. With age-dependent changes in lung morphology, cell integrity, and above all wide variations in severity of acute lung injury, most of the direct (e.g., lavage, MAS) or direct-to-indirect acute lung injury protocols are able to elicit at least some of these responses which may be even more pronounced and more diverse in multiple-hit models.

Direct lung injury in newborn animals aims at alveolar epithelial damage, alveolar edema, generation of hyaline membranes, disruption of the alveolar basal membrane, epithelial-to-mesenchymal transition, PMNL migration, induced macrophage activity, cytokine storm, protease and phospholipase activation, coagulation abnormalities, oxidative stress, and increased production of antioxidants. As prognostic biomarkers indicating increased mortality in human ARDS patients do not differentiate direct and indirect forms of ARDS [SP-D in serum excepted (Calfee et al., 2015)] it is important to acknowledge that existing neonatal lung injury models almost exclusively represent direct lung injury for reasons of achievement of acuity, stability of the model, and indispensable characteristics of acute lung injury involving clinical features, physiological changes, biological changes, and pathological changes (Matute-Bello et al., 2008). From a practical point of view newborn models of NARDS may therefore profit more from surfactant therapy than the lungs from indirect models as demonstrated in clinical studies with children (Khemani et al., 2019) and adults (Taut et al., 2008). Finally, newborn animal models of NARDS profit from small body sizes/weights in order to evaluate specific therapy modalities, such as surfactant administration in escalating doses, antibody therapies, or modulation of major pro-inflammatory pathways, all of which are subject to R and D and may be extremely expensive. In this regard it is important to be aware of the similarities in NARDS, PARDS, and ARDS (De Luca, 2019), of the impact of the innate immunity, and of the advantage of being able to transfer findings from neonatal to more mature animal models (Schouten et al., 2015).

### Future Direction: Customizing Innate Immunity

In the majority of cases NARDS is elicited by the invasion of PAMPS (pathogen-associated molecular patterns) into the lungs which may be bacteria, meconium, droplets of bile, amniotic fluid, or swallowed blood among others. These pathogens may be bound by macrophages with the help of surface lectins or Toll-like receptors also inducing macrophage activation. The surfactant proteins A and D are collectins and provide a first line defense next to molecules of the ficolin and pentraxin families which act as opsonins together with the complement system. Of paramount significance is the early invasion of PMNL to the site of inflammation (alveolar epithelium, capillary endothelium of the lung) mediated by CAMs (cellular adhesion molecules, interaction with PMNL integrins) and selectins (interaction with carbohydrate ligands). In addition, cytokines, such as TNF-α, IL-1β, and chemokines move PMNL and plasma molecules to the site of inflammation or tissue damage. The clearance of pathogens and cell debris is followed by remodeling and regeneration of pulmonary tissue including epithelial-to-mesenchymal transition (EMT) and the proliferation and mobilization of fibroblasts (being responsible for the rapidly declining C_rs_ (compliance of the respiratory system) after some days of mechanical ventilation).

Limiting damage and repair of lung tissue by the newborn organism's innate immunity without completely uncoupling the means of defense—especially in case of infectious pathogens—seems to be the distinguished task for future research by the use of the piglet lavage/meconium aspiration model. Considering the complexity of the innate immunity as very shortly outlined above, many approaches are possible but should probably tackle major anti-inflammatory pathways instead of single—even important—molecules to overcome the phenomenon of redundant activation of e.g., many cytokines [such as blocking IL-8 by specific antibodies may upregulate IL-8 production in experimental NARDS (Ankermann et al., 2005b)].

### Conclusions

The newborn piglet serves as an excellent, robust animal model to study severe neonatal lung diseases with high mortality. The research of three decades has described a myriad of physiological and immunological parameters of the newborn piglet as one of the best studied animal models ever. Most of the clinical, physiological, biological, and pathological changes in NARDS can be also found in the two well-established models presented here: the meconium aspiration model and the lavage model. While most of the research was conducted in the last decade and has slowed down lately, many new insights into the innate immune system should bring up new treatments to specifically tackle important pro-inflammatory upstream pathways. For the benefit of many newborns with life-threatening nARDS future research on the newborn piglet models may greatly help to conquer new specific treatment modalities.

## Author Contributions

DS and NR collected the physiologic data from three experiments and screened eligible publications for the systematic review. MK wrote the first draft of the manuscript. All authors contributed to a manuscript revision and approved the final version of the manuscript.

### Conflict of Interest

The authors declare that the research was conducted in the absence of any commercial or financial relationships that could be construed as a potential conflict of interest.

## Footnotes

^1^National Heart, Lung, and Blood Institute. *Study Quality Assessment Tools, Quality of Case-Control Studies*. Available online at: https://www.nhlbi.nih.gov/health-topics/study-quality-assessment-tools.

## Abbreviations

AEC: alveolar epithelial cells
Ang-2: angiopoietin 2
ANOVA: analysis of variance
ARDS: (adult) acute respiratory distress syndrome
ASC: apoptosis-associated speck-like protein containing a caspase recruitment domain
aSMase: acid sphingomyelinase
AT III: antithrombin III
BALF: broncho-alveolar lavage fluid
(S/D)BP: (systolic/diastolic) blood pressure
BPD: broncho-pulmonary dysplasia
C: control group
CD14/18/61: cluster of differentiation 14/18/61
CI: confidence interval
C3/5a: complement factor 3/5 activated
DOPG: dioleoyl-phosphatidylglycerol
EMT: epithelial-to-mesenchymal transition
EVLWI: extra-vascular lung water index
F_i_O_2_: fraction of inspired oxygen
FRC: functional residual capacity
G-CSF: granulocyte colony stimulating factor
GBS: group B streptococci (*Streptococcus agalactiae*)
HC: healthy control group: piglets not subject to lung injury
HI: heart index
HR: heart rate
HVR: hypervariable region (of Toll-like receptor 4)
IFN(-γ): interferon (γ)
IL-1(α/β): −6, −8, interleukin 1(α/β), 6, 8
IL-1 ra: interleukin 1 receptor agonist
IRDS: respiratory distress syndrome of the premature infant
ITBI: intra-thoracic blood volume index
KC: murine functional chemokine analog of IL-8
LPS: lipopolysaccharide
LTB_4_: leukotriene B_4_
MAP: mean airway pressure
MCP-1: monocyte chemotactic protein 1
MD-2: lymphocyte antigen 96
MIP-2: macrophage inflammatory protein 2
MMP-1/2/8/9: matrix metalloproteinase 1/2/8/9
MPO: myeloperoxidase
NARDS: neonatal acute respiratory distress syndrome
NF-κB: nuclear factor κB
NLRP(3): inflammasome nucleotide-binding domain—leucine rich repeat- containing protein-(3)
NO: nitric oxide
OI: oxygenation index (MAP ^*^ %O_2_/PaO_2_)
PaCO_2_: partial pressure of carbon dioxide
PAI-1: plasminogen activator inhibitor 1
PAMP: pathogen-associated molecular pattern
PAP: pulmonary arterial pressure
PARDS: pediatric acute respiratory distress syndrome
PC: phosphatidylcholine
PEEP: positive end-expiratory pressure
PIM: pulmonary intravascular macrophages
PIP: peak inspiratory pressure
PMNL: polymorpho-nuclear leukocytes
POPG: palmitoyl-oleoyl-phosphatidylglycerol
PVRI: pulmonary vascular resistance index
rhCC10: recombinant human Clara Cell protein 10
ROS: reactive oxygen species
R_rs_: resistance of the respiratory system
sC_rs_: specific compliance of the respiratory system
SA/LA: small/large (surfactant) aggregates
sC5b-9: soluble complement factor 5b-9, membrane attack protein
SD: standard deviation
sICAM-1: soluble intercellular adhesion molecule 1
SP(-A/B/D): surfactant protein (-A/B/D)
sPLA_2_: soluble phospholipase A_2_
sRAGE: soluble receptor for advanced glycation end product
SVI: stroke volume index
SVV: stroke volume variation
SVRI: systemic vascular resistance index
T: treatment group
TAT: thrombin antithrombin complex
TGF-β: transforming growth factor β
TLR4: Toll-like receptor 4
TNF-α: tumor necrosis factor α
TTN: transient tachypnea of the newborn
VEI: ventilation efficiency index (3,800/(PIP-PEEP^*^f^*^PaCO_2_))
V_A_: alveolar portion of the tidal volume
V_T_: tidal volume
vWF: von Willebrand factor.
